# An Ephemeral Sexual Population of *Phytophthora infestans* in the Northeastern United States and Canada

**DOI:** 10.1371/journal.pone.0116354

**Published:** 2014-12-31

**Authors:** Giovanna Danies, Kevin Myers, María F. Mideros, Silvia Restrepo, Frank N. Martin, David E. L. Cooke, Christine D. Smart, Jean B. Ristaino, Abby J. Seaman, Beth K. Gugino, Niklaus J. Grünwald, William E. Fry

**Affiliations:** 1 School of Integrative Plant Science, Plant Pathology and Plant-Microbe Biology Section, Cornell University, Ithaca, New York, United States of America; 2 Department of Biological Sciences, Universidad de los Andes, Bogotá, Colombia; 3 United States Department of Agriculture – Agriculture Research Services (USDA-ARS), Salinas, California, United States of America; 4 The James Hutton Institute, Invergowrie, Dundee, United Kingdom; 5 Department of Plant Pathology and Plant-Microbe Biology, Cornell University, Geneva, New York, United States of America; 6 Department of Plant Pathology, North Carolina State University, Raleigh, North Carolina, United States of America; 7 New York State Integrated Pest Management Program, Cornell Cooperative Extension, Geneva, New York, United States of America; 8 Department of Plant Pathology and Environmental Microbiology, The Pennsylvania State University, University Park, Pennsylvania, United States of America; 9 Horticultural Crops Research Laboratory, USDA Agricultural Research Service, Corvallis, Oregon, United States of America; 10 Department of Botany & Pant pathology and Center for Genome Biology and Biocomputing, Oregon State University, Corvallis, Oregon, United States of America; Agriculture and Agri-Food Canada, Canada

## Abstract

*Phytophthora infestans*, the causal agent of late blight disease, has been reported in North America since the mid-nineteenth century. In the United States the lack of or very limited sexual reproduction has resulted in largely clonal populations of *P. infestans*. In 2010 and 2011, but not in 2012 or 2013, 20 rare and diverse genotypes of *P. infestans* were detected in a region that centered around central New York State. The ratio of A1 to A2 mating types among these genotypes was close to the 50∶50 ratio expected for sexual recombination. These genotypes were diverse at the *glucose-6-phosphate isomerase* locus, differed in their microsatellite profiles, showed different banding patterns in a restriction fragment length polymorphism assay using a moderately repetitive and highly polymorphic probe (RG57), were polymorphic for four different nuclear genes and differed in their sensitivity to the systemic fungicide mefenoxam. The null hypothesis of linkage equilibrium was not rejected, which suggests the population could be sexual. These new genotypes were monomorphic in their mitochondrial haplotype that was the same as US-22. Through parentage exclusion testing using microsatellite data and sequences of four nuclear genes, recent dominant lineages US-8, US-11, US-23, and US-24 were excluded as possible parents for these genotypes. Further analyses indicated that US-22 could not be eliminated as a possible parent for 14 of the 20 genotypes. We conclude that US-22 could be a parent of some, but not all, of the new genotypes found in 2010 and 2011. There were at least two other parents for this population and the genotypic characteristics of the other parents were identified.

## Introduction

*Phytophthora infestans*, a plant pathogenic oomycete, is a major constraint to potato and tomato production globally [1]. This pathogen reproduces both sexually and asexually, the former only when both mating types, A1 and A2, occur in the same location. Sexual reproduction is apparently uncommon worldwide with the exception of some regions such as central Mexico and northern Europe [1]–[3]. Sexual reproduction results in the production of spores (oospores) [4] resistant to environmental extremes, which serve as a source of primary inoculum when present in the soil [5]. Oospores are able to survive in a dormant state for several years until conditions are optimal for the spores to germinate and cause infection [6]. In the United States there is no evidence for continual sexual reproduction even though both mating types exist. This apparent lack of widespread sexual reproduction has resulted in clonal populations of *P. infestans*. A clonal lineage is a descendant from a single individual and variation within a lineage arises by mutation or mitotic recombination [7], [8]. The diverse clonal lineages of *P. infestans* present in the United States have likely been introduced via migrations [9], [10].

Historically, clonal lineages of *P. infestans* have been defined based on mating type, mitochondrial haplotype, nuclear DNA fingerprint patterns, and allozyme genotype [9]. More recently, microsatellite markers have been utilized to characterize USA populations [11], [12]. Individuals within a lineage have the same multi-locus genotype (MLG) with only minor variation. Clonal lineages in the United States have typically been quite distinct, commonly defined using a subset of the markers identified above. Individual isolates within a clone also share phenotypic traits such as fungicide sensitivity, host preference, and aggressiveness [10], [13]–[18]. However, mutations at pathogenicity loci occur rapidly [10].

Twenty-four diverse genotypes or clonal lineages of *P. infestans* have been detected and have been dominant in the past 40 years in the United States [19]. Several lineages of *P. infestans* may coexist in the United States in any particular year but epidemic populations have typically been composed of one or rarely a few lineages. In the summers of 2010 and 2011 (but not in 2012 or 2013), greater diversity was detected in west-central New York State than had been observed in the entire United States in the previous ten years.

The primary objective of this study was to determine whether the diverse population of *P. infestans* detected around west-central New York State in 2010 and 2011 was a result of sexual reproduction. Genetic markers with different mutation rates (nuclear and mitochondrial genes) were used to address the following two working hypotheses: (1) These rare and diverse genotypes of *P. infestans* are the outcome of one or more recombination events; and (2) if so, one of the currently dominant clonal lineages in the USA might be a parental genotype. The hypothesis that these genotypes might represent a sexual population was not rejected. Parental exclusion tests failed to eliminate lineage US-22 as a potential parent for most of the NYS-2010/11 population. Other parents must be involved, and the genotypic characteristics of these other parents are inferred.

## Materials and Methods

### Isolates

Isolates used in this study were those obtained from samples submitted by persons with extension responsibilities. Samples were infected tomato or potato leaflets showing typical late blight symptoms. The samples were submitted voluntarily by the owners of the affected plants. The domestic permit for submission of samples was APHIS permit P526P-13-03974 (or its predecessor) and the international permit for submission of samples was APHIS permit P526P-14-00763 (or its predecessor). In total there were 59 isolates including 20 USA reference isolates and 39 isolates detected in an area that centered around west-central New York State in 2010 and 2011. The population detected in and around New York State in 2010 and 2011 is referred to as the NYS-2010/11 population and the individuals are referred to as GDT-01 through GDT-20 (S1 Table). Cultures were maintained and DNA was extracted as previously described [13]. Different sets of genetic markers, an allozyme test using the glucose-6-phosphate isomerase, a restriction fragment length polymorphism assay using a moderately repetitive DNA probe RG57, mitochondrial haplotyping, 12 microsatellite loci, and four nuclear gene sequences as well as the mating type of each isolate were used to determine the isolate's genotype.

### Initial diversity assays

*Mating type*. Mating type was determined by pairing an unknown isolate with a known isolate of *P. infestans*, either A1 mating type (US970001 US-17 genotype) or A2 mating type (US040009, US-8 genotype), on rye B [20] or pea [21] agar media. Petri plates were kept at 20°C for 10–14 days. The hyphal interface of the two colonies was investigated microscopically using 125X magnification. Isolates that formed oospores at the interface with the known A1 isolate were designated A2 and those that formed oospores with the known A2 isolate were designated A1. The known isolates (A1 and A2) were paired as positive controls, while negative controls consisted of pairing the known isolates with themselves (same mating type).

#### Glucose-6-phosphate isomerase

Mycelia and/or sporangia obtained from cultures grown on rye B, pea agar or from infected leaflets were used to determine *glucose-6-phosphate isomerase* (*GPI*) allozyme genotypes. Analyses were carried out using cellulose acetate electrophoresis as previously described [22]. At least one reference isolate representing US-1 (SA960008), US-8 (US040009), and/or US-17 (US970001) was included in all *GPI* analyses.

#### DNA extraction and RFLP analysis with probe RG57

DNA extractions and subsequent restriction fragment length polymorphism (RFLP) analysis with the RG57 DNA probe were performed using a method modified from Goodwin et al. [23]. Southern blot analysis was conducted using the Amersham gene images AlkPhos direct labeling and detection system (GE Healthcare) according to the manufacturer's instructions. The US-1 (SA960008) reference isolate was used in RG57 analyses. Presence or absence of known fingerprint fragments was scored visually.

#### Mefenoxam sensitivity assay

Mefenoxam sensitivity of isolates was assessed as described previously by Therrien et al. [24], except that mefenoxam was used in place of metalaxyl. Isolates were grown on pea agar amended with Ridomil Gold SL (Syngenta, Greensboro, NC) such that the final concentrations of the active ingredient (mefenoxam) were 0, 5, or 100 µg ml^−1^. Mycelial plugs (8 mm diameter) were obtained from actively growing cultures, transferred to the test plates and incubated for approximately 10 to 12 days, or until growth on the control mefenoxam plate (0 µg ml^−1^) was approximately 75 to 90% of the diameter of the petri plate. Assessment of mefenoxam sensitivity was determined on the basis of radial growth of cultures grown on plates amended with mefenoxam (5 or 100 µg ml^−1^) compared to non-amended controls. Growth on mefenoxam-amended plates, 5 and 100 µg ml^−1^, was represented as a proportion of the growth on the non-amended control plates.

#### Mitochondrial haplotyping

Mitochondrial haplotype was determined following the protocol reported by Martin et al. [25]. This protocol was designed to determine the smallest number of loci needed to classify mitochondrial haplotypes for *P. infestans*. In total five loci were sequenced. The first locus included the *rpl5-rns* region that includes the downstream part of P3 and most of P5. The second locus included regions *rns-cox2*, as well as *orf79*. The third locus included regions *cox1*-*nad9*, including *atp-9* and the downstream half of the P4 region. The fourth locus included *nad3-nad5*. The fifth locus included regions *nad6-nad4L* as well as the upstream half of the P6 region. This procedure has allowed the identification of at least 36 different mitochondrial haplotypes in *P. infestans*. The first step of this mitochondrial haplotyping protocol was to sequence the second and fifth loci for all isolates. This would allow the discrimination of at least 27 haplotypes. Based on the results obtained from sequencing loci two and five, it was only necessary to sequence locus three in all isolates to distinguish between haplotypes.

### Multiplex microsatellite marker analysis

Twelve microsatellite loci previously demonstrated to reveal polymorphisms [26] were genotyped for all isolates. Genotyping was conducted using the QIAGEN Type-it Microsatellite PCR Kit (QIAGEN, Cat. No. 206243). Amplifications were done as described by the manufacturer. PCR conditions were: 95°C for 5 min followed by 30 cycles of 95°C for 30 s, 58°C for 90 s, and 72°C for 20 s, and a final extension at 60°C for 30 min. PCR products were analyzed on an ABI 3730xl capillary system with POP-7 Polymer (ABI, PN 4335615). PCR amplicons were compared with a set of size standards and alleles were scored accordingly [26]. At least one reference isolate representing US-8 (US100028), US-11 (US110028), US-22 (US090042), US-23 (BL2009P4) and/or US-24 (ND822Pi) was included in all microsatellite analyses.

### Analyses using microsatellite data

he R package *Poppr 1.0.5* [27], which allows analysis of populations with mixed modes of reproduction (sexual and asexual) was used to analyze the microsatellite data. The 59 isolates were arbitrarily clustered into three groups according to their occurrence over time in the United States. Group one contained eight isolates including lineages of *P. infestans* that had been prevalent in the United States at one time, but not for the past 10 years (US-1, US-6, US-7, US-12, US-14, US-16, US-17, and US-19). Group two contained 12 isolates including lineages of *P. infestans* that were dominant over the past five years (US-8, US-11, US-22, US-23 and US-24) or that were first described during the past five years in the United States (US-20 and US-21). Group three contained the 39 NYS-2010/11 *P. infestans* isolates that are the focus of this study (GDT-01 to GDT-20).

To detect signs of linkage disequilibrium across the microsatellite loci, a standardized index of association that corrected for the number of loci (*řd*) [28] was calculated using clone corrected data.

Two different types of analyses were conducted to determine if the GDT isolates cluster as a single population and to observe if any of the previously known clonal lineages cluster with them. Population structure was inferred using the program *structure* 2.3 [29] by testing the number of population clusters (K) between 1 and 10 using the admixture model [30]. A total of 10 independent runs of 100,000 iterations with burn-in period of 50,000 MCMC iterations were conducted. The results from *structure* were post-processed using *Structure Harvester* [31]. The *ΔK* method according to Evanno et al. [32] was used to evaluate the rate of change in the log probability of data between successive *K* values to infer the number of populations.

To visually assess between-population differentiation, and assess the contribution of individual alleles to population structuring, a discriminant analysis of principal components (DAPC) was done using the *adegenet* package for R [33]. This multivariate method extracts information from genetic data and identifies genetic clusters using the *k*-means clustering algorithm based on the Bayesian Information Criterion [34]. This clustering algorithm finds a given number of groups that maximizes the variation between groups, and describes the relationships between these clusters.

### Nuclear gene sequencing

Genes known to be polymorphic within *P. infestans* [35]–[38] were chosen for sequencing (Table 1). PCR conditions for nuclear loci were the following, in a final volume of 20 µl: 1X PCR buffer with a final MgSO_4_ concentration of 2 mM, 200 µM dNTPs, 0.5 µM of each primer, one unit of Platinium *Taq* High Fidelity (Invitrogen), and ∼10 ng template DNA. Thermal cycling protocols used an initial denaturation step at 94°C for two minutes; 35 cycles of 94°C for 30 sec, locus-specific annealing temperature for 30 seconds (Table 1), 68°C extension for 45 seconds; and a final extension at 68°C for 5 minutes.

**Table 1:** Nuclear loci sequenced, primers, and amplification conditions used in this study.

Locus	Primer name	Primer sequence (5′ – 3)	T_a_ a	Reference
*PITG_11126* (Conserved Hypothetical Protein)	PITG11126_F1	GGGGACTTCGCTGTTTGTTA	59.0°C	[38]
	PITG11126_R1	ATGTTCATGTACGGCTGACG		
*PUA* (Conserved Hypothetical Protein)	PUA_F	AGGTCAAGTCCTCGCAGCAG	67.0°C	[35]
	PUA_R	AGGTCGTCRCCMAAGTG		
*β –tubulin*	Btub_F1	GCCAAGTTCTGGGAGGTCATC	58.4°C	[36]
	Btub_R1	CCTGGTACTGCTGGTACTCAG		
*TRP1* (N-(5′-phosphoribosyl)anthranilate isomerase indole-3-glycerol-phosphate synthase)	TRP1_F3	GGGTAACATCCTGGAGGAGA	63.0°C	[37]
	TRP1_R3	TCGTACTTGACCACGTCTGC		

a Annealing temperature of primers for PCR.

PCR products were visualized on a 1% agarose gel to confirm amplicon size. An enzymatic purification protocol was used following the manufacturer's instructions (ExoSAP-IT, Affymetrix), and products were sequenced using BigDye terminator chemistry, and analyzed on an ABI 3730 instrument (Applied Biosystems) at the Cornell University Sequencing Core Facility. ABI trace files were analyzed using Geneious Pro v4.8.5 [39]. Sequence alignments were generated using MUSCLE [40] with default settings. Bases with overlapping peaks in the electropherograms were considered heterozygous and coded according to IUPAC convention. Sequences were deposited to GenBank under the accession numbers [KM249146-KM249175].

### Analyses using nuclear gene sequences

Haplotype phase of nuclear gene sequences with two or more heterozygous sites were determined by cloning PCR products for a subset of genotypes and sequencing inserts. Cloning of PCR products was done using the TOPO TA Cloning Kit for Subcloning, with One Shot TOP10 chemically competent *Escherichia coli* cells (Invitrogen). Isolates cloned were SA960008 (US-1) and US100032 (GDT-15) for *PITG_11126*; SA960008 (US-1), Coffey7629 (US-6), US100019 (GDT-19) and US110040 (US-23) for *PUA*; US100033 (GDT-16) for *β-tubulin*; and SA960008 (US-1), US940494 (US-12), US050007 (US-11), and US110040 (US-23) for *TRP1*. Polyploids were identified (US-1, US-11, and US-23) and all sequences were included in our downstream analyses. Nucleotide diversity indices and the polymorphic level for the four nuclear genes studied were calculated using DNAsp v. 5.10.01 [41]. To test for neutral evolution, Tajima's D [42] was calculated using DnaSP v. 5.10.01.

Jmodeltest [43] was used to estimate a nucleotide substitution model. Maximum likelihood (ML) gene trees were inferred using PhyML [44], implemented in the South of France bioinformatics platform (http://www.atgc-montpellier.fr/phyml/), using the substitution model selected by jmodeltest (K80 for PITG_11126, TN93 for TRP1, JC69 for *β*-tubulin and PUA). The transition/transversion ratio, proportion of invariable sites, and gamma distribution parameter were estimated from the data in PhyML using 6 rate categories. Data sets were bootstrapped using 1000 samples.

Gene trees were also inferred using MrBayes [70], implemented in CIPRES Science Gateway [45]. The same nucleotide substitution model was used as for PhyML. One million MCMC generations were run, using a sample frequency of 500 generations and a burn-in of 25% of the total run. Two runs using four chains each (one cold and three heated chains) were performed. The default priors were used.

### Parentage exclusion analysis

A visual parentage exclusion analysis [46] was possible given that out of the 59 isolates (including USA reference isolates as well as GDT isolates) there were 37 unique *P. infestans* genotypes after clone correcting. This analysis is based on the fact that a particular offspring has two alleles for each autosomal marker corresponding to one from each of its progenitors. Based on this analysis, a genotype was excluded as a potential parent of one of the NYS-2010/11 isolates, if neither of the alleles present at a particular locus in the candidate parent was present in the candidate progeny (a flow diagram with a chain of logic is shown on S1 Fig.). Both microsatellite alleles and nuclear gene sequences were used for this analysis.

## Results

**Figure 1:**
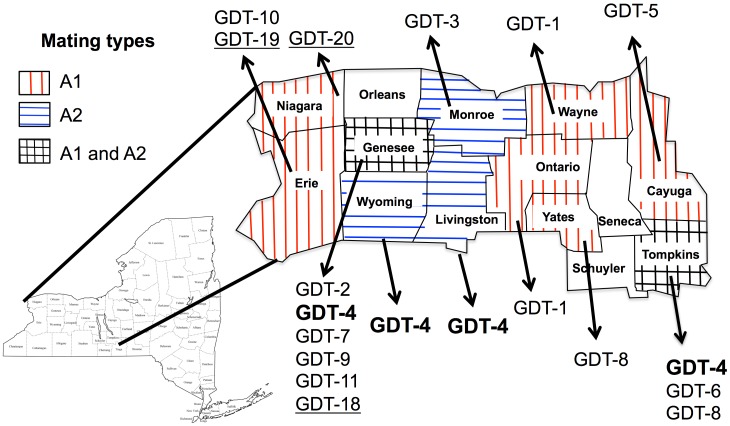
Spatial occurrence of the NYS-2010/11 population of *Phytophthora infestans* detected in western New York State. Genotypes that are underlined are those detected in 2010 all other genotypes were detected in 2011. Genotypes shown in bold are those that were found in several counties (GDT-01, GDT-04, and GDT-08). In New York State we detected only A1 individuals in six counties, and only A2 individuals in another three counties. However, both A1 and A2 individuals were reported from yet two other counties. Because of our limited sample size, we cannot conclude with certainty that both mating types were not present in counties where only a single mating type was detected.

In 2010 and 2011 we confirmed that there were individuals of *P. infestans* of opposite mating type from west-central New York and surrounding areas (Figure 1). We detected only A1 individuals in some counties, and only A2 individuals in other counties. However, both A1 and A2 individuals were reported from yet other counties. Thus, it was clear that this outbreak differed from most recent outbreaks in the United States in that there was a large region in which both mating types were admixed.

**Figure 2:**
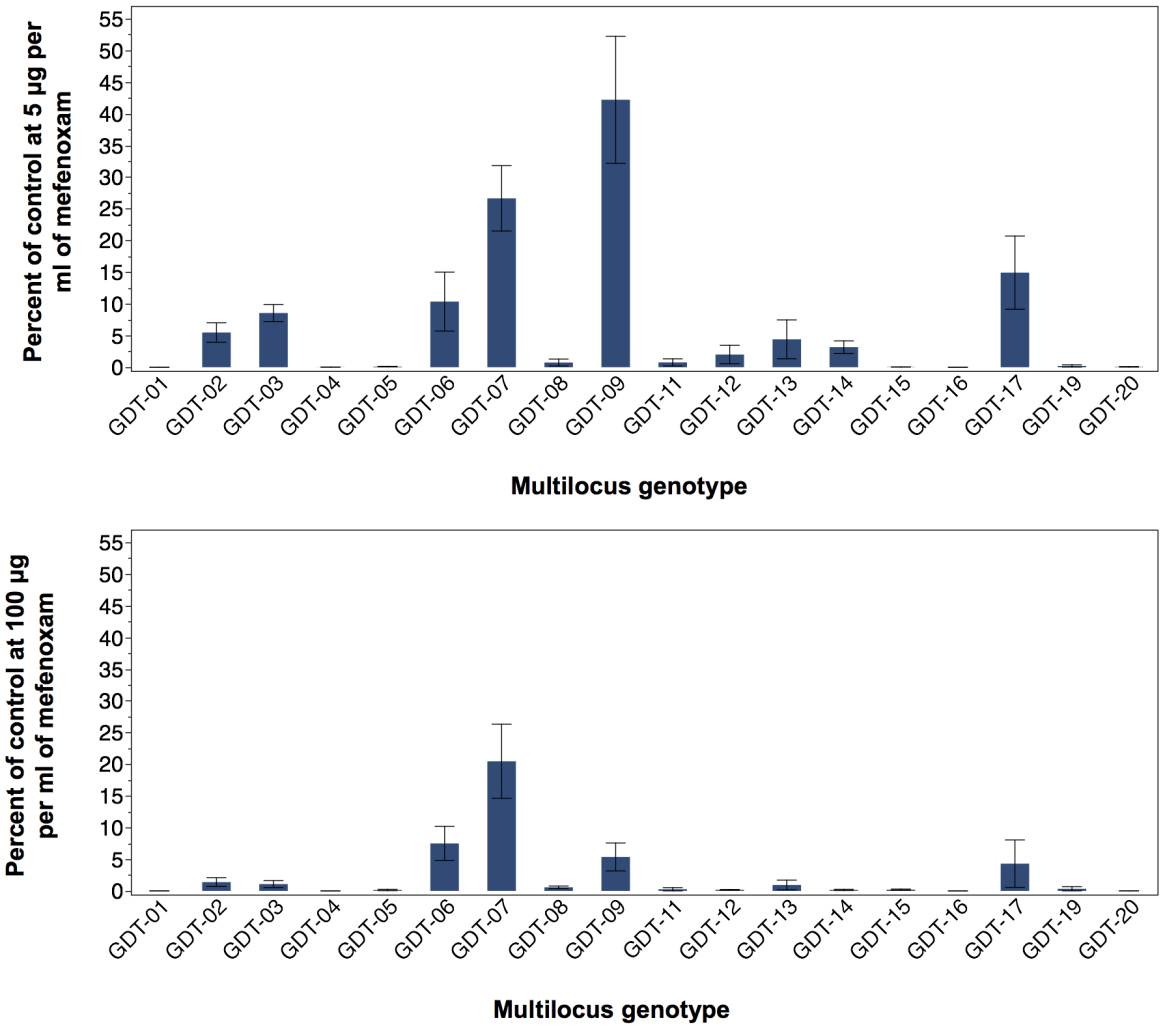
Response of *Phytophthora infestans* isolates to mefenoxam. Relative growth (as percentage of control) at 5 µg ml^−1^ (top) and 100 µg ml^−1^ (bottom) relative to control 0 µg ml^−1^ is presented. Sample sizes for each multilocus genotype can be found in S1 Table.

Further analyses confirmed that these individuals constituted a diverse population. These genotypes displayed different banding patterns for the allozyme *glucose-6-phosphate isomerase* locus (S2 Table), showed different banding patterns at four loci in a restriction fragment length polymorphism assay using the moderately repetitive and highly polymorphic probe RG57 (S2 Table), differed in their microsatellite profiles (S3 Table), were polymorphic for four different nuclear genes (S4, S5, S6, S7 Tables) and differed in their sensitivity to the systemic fungicide mefenoxam (Figure 2). However, they were monomorphic for their mitochondrial haplotype (S2 Table). The 39 NYS-2010/11 isolates represented 20 distinct multi-locus genotypes (MLGs). Based on their genotypic profile these new and diverse genotypes were named GDT-01 to GDT-20. In addition to individuals from NY, there were individuals from Ohio, Pennsylvania and Ontario, Canada that also appeared to belong to this population (S1 Table). There were cases, where the same genotype was found in several New York state counties (Figure 1).

### Population genetic analyses and recombination tests using microsatellite markers

In total, 50 microsatellite alleles were detected in the entire collection of genotypes. The number of alleles per locus ranged from two (for locus Pi04) to eight (for PiG11 and PinfSSR4) (S8 Table). Using the microsatellite loci, we tested the different groups against the null hypothesis of sexual reproduction by measuring the extent of linkage equilibrium across the microsatellite loci. When all isolates were analyzed together this null hypothesis was rejected (*P = 0.001*). This null hypothesis was rejected also for Group 1 (old isolates) alone (*P = 0.001*) and for Group 2 isolates (lineages that have been dominant in the United States over the past five years or those that have been detected for the first time during the past five years) alone (*P = 0.002*). However, for the NYS-2010/11 isolates (Group 3), we failed to reject the null hypothesis (*P = 0.1490*). Thus this group seemed to have characteristics of a sexually reproducing population.

### Genetic structure analysis

The population structure based on microsatellite data consisted of two distinct clusters (S2 Fig.) and the *ΔK* analysis indicated that *K* = 2 was most likely the correct minimum number of clusters. Interestingly, US-22 clustered with the NYS-2010/11 population (S2 Fig.). S2 Fig., shows the results for *K* = 2 to *K* = 5. At *K*≥3, population subdivision for the USA reference isolates was large relative to population subdivision for the GDT isolates and lineage US-22.

**Figure 3:**
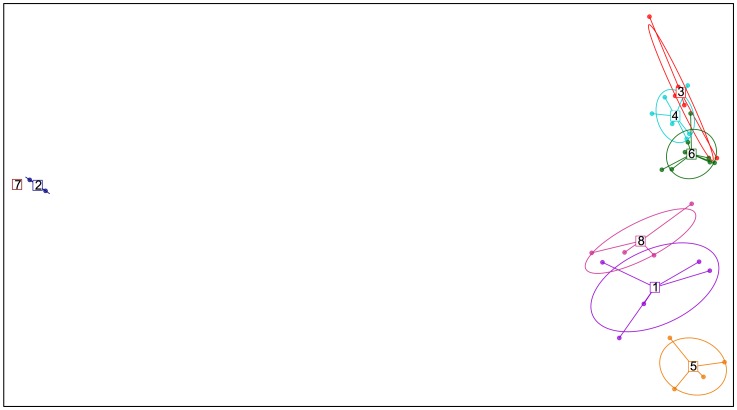
Discriminant Analysis of Principal Components (DAPC) using 12 microsatellite loci. This scatterplot shows the first two principal components of the DAPC of *Phytophthora infestans* genotypes found in the United States. Groups are shown by different colors and inertia ellipses, while dots represent individual strains. Cluster 1 includes lineages US-6, US-7, US-11, US-12, and US-16; Cluster 2 includes isolates in lineage US-23; Cluster 3 includes lineages GDT-02, GDT-07, GDT-13, GDT-18, and GDT-20; Cluster 4 includes lineages GDT-03, GDT-04, GDT-08, GDT-08.1, GDT-14 and GDT-15; Cluster 5 includes lineages US-8, US-14, US-20, and US-24; Cluster 6 includes lineages GDT-05, GDT-06, GDT-09, GDT-10, GDT-11, GDT-12, GDT-16, GDT-17, and GDT-19; Cluster 7 includes lineages US-1; and Cluster 8 included lineages US-17, US-19, US-21 and GDT-01.

In general, the Discriminant Analysis of Principal Components (DAPC) is in agreement with the results obtained using *structure* (Figure 3). Based on the Bayesian Information Criterion, the number of clusters was eight (S3 Fig). Most of the isolates within the NYS-2010/11 population (19 out of 20) clustered together with US-22. GDT-01 was grouped on a separate cluster along with lineage US-17, US-19, and US-21.

### Population genetic analyses of nuclear loci

Nucleotide diversity was low for the four nuclear genes studied (Table 2). The nuclear gene *PUA* showed the highest number of segregating sites as well as the highest number of haplotypes (Table 2 and S5 Table). Tajima's D was not significant indicating that all genes were evolving neutrally (0.51174, −0.75042, 1.01645, and −0.80032 with *p>0.1* for genes *PITG_11126*, *PUA*, *β-tubulin*, and *TRP1* respectively). Both negative and positive Tajima's D values were observed, yet these differences are minor and not distinguishable from noise. The four nuclear genes did not resolve the same topologies (S4 Fig.). Clusters (defined according to the occurrence of *P. infestans* isolates over time in the United States) showed no clear grouping. Conflict among the gene trees is likely due to recombination among individuals. Even low levels of recombination leads to conflict among gene genealogies [47].

**Table 2:** Summary statistics of loci *PITG_11126*, *PUA*, *β-tubulin* and *TRP1* from *Phytophthora infestans* in North America.

Locus	Number of individuals^a^	Length (bp)	Segregating sites	Genetic variability (Segregating sites/Length)	π^b^	θ Site^c^	Number of haplotypes	HD^d^
*PITG_11126*	40	776	6	0.00773	0.00190	0.00156	5	0.631
*PUA*	48	609	9	0.01478	0.00226	0.00320	9	0.520
*β-tubulin*	41	883	3	0.00340	0.00106	0.00068	4	0.517
*TRP1*	48	824	6	0.00728	0.00092	0.00142	8	0.435

The number of individuals per gene is different because for some genes more than one isolate per genotype was sequenced.

a In some cases multiple isolates per multilocus genotype were sequenced.

b π = Nucleotide diversity (per site).

c θ site = Watterson's Theta (per site).

d HD = Haplotype diversity.

### Parentage Analysis

If the NYS-2010/11 population represents a population that resulted from recent sexual reproduction, it would be interesting to determine the potential parents. We assumed that any of the lineages that have been dominant in the United States over the past five years or those that have been detected for the first time during the past five years (Group 2) could be potential parents for the GDT isolates. Therefore we conducted a parentage exclusion test using microsatellite data. Based on this analysis, a potential parent of a candidate progeny was excluded if neither of the alleles present at a particular locus in that potential parent was present in the candidate progeny. For example, clonal lineage US-8 has alleles 108 and 112 for locus D13. Neither of these alleles was present in any of the NYS-2010/11 isolates. Thus, US-8 was excluded as a potential parent. Based on similar analyses it was possible to exclude lineages US-20, US-21, US-23, and US-24 as possible parents for the NYS-2010/11 isolates.

Lineages US-22 and US-11 remained as potential parents for at least some (but not all) of the NYS-2010/11 isolates. For example, US-22 was excluded as a parent for GDT-06, based on locus PinfSSR4; it was excluded as a potential parent for GDT-11 based on locus PinfSSR11 and it was excluded as a parent for GDT-12 based on two microsatellite loci PinfSSR4 and PinfSSR11 (S9 Table). Microsatellite data did not exclude US-22 as a parent for the other 17 GDT isolates. On the other hand, after parentage exclusion analysis using microsatellite data, clonal lineage US-11 remained as a potential parent only for seven of the twenty NYS-2010/11 isolates, GDT-03, GDT -04, GDT -06, GDT -09, GDT -10, GDT -14, and GDT -15 (S10 Table).

The diversity in the nuclear genes studied enabled further analysis of potential parentage of the NYS-2010/11 population. Using sequence analysis of the gene coding for the hypothetical protein PITG_11126, it was possible to exclude three additional GDT genotypes (GDT-04, GDT-05, and GDT-09) as progeny of US-22 (S4 Table). Analysis of the other three genes used in this study (*PUA*, *β-tubulin* and *TRP1*) provided data that were consistent with our hypothesis that US-22 is a possible parent for some of the GDT isolates (S5, S6, S7 Tables). Analysis of nuclear genes was also applied to US-11 as a potential parent of the GDT isolates. Using sequence analysis of the genes coding for the hypothetical proteins *PITG_11126* and *PUA*, it was possible to exclude 14 GDT isolates as potential progeny of US-11. However, the six genotypes that remained as potential progeny of US-11 (GDT-02, GDT-08, GDT-12, GDT-13, GDT-18, and GDT-20) had been excluded as potential progeny of US-11 based on the microsatellite data.

If US-22 is one of the parents for at least some of the isolates within the NYS-2010/2011 population, it is possible to predict the banding pattern for the *glucose-6-phosphate isomerase*, the mating type and the microsatellite profile for the other putative parent(s). Assuming that US-22 is one of the parents, at least one of the other parent(s) must be A1 mating type. The other parent(s) must have an 111 allele for the *glucose-6-phosphate isomerase*, and must provide the following microsatellite alleles: an allele 110 for the D13 locus, an allele 189 for the Pi70 locus, an allele 225 for the Pi4B locus, an allele 258 for the Pi02 locus, alleles 284 and 288 for the PinfSSR4 locus and an allele 355 for the PinfSSR11 locus. These predictions are shown in S11 Table. In the same way, it is possible to predict the potential genotypes of loci *PITG_11126*, *PUA*, *β-tubulin* and *TRP1* of putative parent(s) for the GDT isolates when assuming that lineage US-22 is one of the parental genotypes for these isolates (S12 Table).

The mitochondrial haplotype data were interesting but not conclusive regarding parentage. Clonal lineage US-22 has the same mitochondrial haplotype (H20) as all of the GDT isolates.

## Discussion

The genetic characteristics of the ephemeral population of *P. infestans* detected in the west-central region of New York State in 2010 and 2011 are consistent with a recombinant population. Greater diversity was detected in that region during each of 2010 and 2011 than had been observed in the entire United States in the previous ten years. The sampling strategy or sampling intensity during 2010 and 2011 did not differ from other years. The number of samples received by our laboratory in 2010 and 2011 was 51 and 137, respectively. In 2012 and 2013 the number of samples received was 237 and 274, respectively. Thus, the diversity observed during 2010 and 2011 cannot be explained by an increase in the number of samples received and analyzed.

Our analyses of the NYS-2010/11 population are retrospective because they occurred after we became aware that these isolates were indeed unusual. It is challenging to obtain a truly random sample of *P. infestans*, given that late blight outbreaks occur sporadically and are typically clonal in the USA. Selection and/or drift may have played an important role prior to our analyses and may be an explanation for the disappearance of these genotypes after 2011. As a result we cannot regard these isolates as a comprehensive sample of a segregating population. However, their occurrence in a relatively small geographical area is unusual given the population structure of *P. infestans* in the USA. Only one other population with similar characteristics has been reported in the USA – in the Pacific Northwest in the late 1990s [48]. This population also had characteristics of a recombinant population [48].

The NYS-2010/11 population displayed diverse banding patterns for the allozyme *glucose-6-phosphate isomerase*, showed different banding patterns in a restriction fragment length polymorphism assay using the moderately repetitive and highly polymorphic probe RG57, differed in their microsatellite profiles, were polymorphic for four different nuclear genes and differed in their sensitivity to the systemic fungicide mefenoxam. The ratio of mating types among the NYS-2010/11 genotypes was close to the 50∶50 ratio expected for sexual recombination.

The population structure of *P. infestans* in the USA is dramatically different from that in Sweden [49] and central Mexico [2] where sexual reproduction is a very common and significant factor in the ecology of *P. infestans*. The sexual population in Sweden is very recent (occurring in the latter part of the 20th century) and contributes to earlier and more devastating epidemics than before the occurrence of that population [49]. The sexual population in central Mexico is very old, because this location is the likely center of origin of *P. infestans* [50]. It seems very likely that sexual recombination is still very rare and not a persistent contributor to the ecology of *P. infestan*s in the USA. However, the documentation now of two likely recombinant populations ([48]; and this study) indicate that sexual recombination in the USA is certainly possible and there is no reason to believe it will not happen again.

The genetic structure analyses done using *structure* and a discriminant analysis of principal components showed that the GDT isolates were grouped closely together and US-22 clustered within the GDT isolates. The index of association tested using microsatellite data failed to reject the hypothesis that the NYS-2010/11 individuals were a recombinant population. There are two possible scenarios that could explain this occurrence: 1) the recombination event(s) happened in or near west-central New York State or 2) the recombination event(s) took place somewhere outside New York State and the progeny from this event migrated as a cohort to central New York State, presumably on potato tubers or tomato transplants. We have no definite evidence of the location of the recombination event(s), or if the oospore population still exists there. The fact that these strains have not been detected since 2011 is excellent news. They may have all died out. However, the fact that this population existed indicates that sexual recombination in the United States is a current possibility, and may happen again.

Our data suggest that clonal lineage US-22 could be a parent of some, but not all, of the new genotypes detected in 2010 and 2011. This is consistent with the fact that US-22 was the dominant lineage throughout the eastern United States in 2009 [19]. In 2009 US-22 represented approximately 90% of the samples received and analyzed by our lab [19]. At least two more parents are expected based on the nuclear gene sequences in the NYS-2010/11 population. This is because some isolates within the NYS-2010/11 population were homozygous for a site where US-22 was homozygous for a different nucleotide.

An additional hypothesis is that US-22 is a sibling of the GDT isolates, rather than a parent. Although we cannot definitively reject this hypothesis, at least two lines of reasoning support parentage rather than sib status for US-22 relative to the NYS-2010/11 population. First, US-22 was very widely distributed across the entire eastern part of the USA in 2009, so chronologically, US-22 was detected before the GDT isolates [19], [51]. Second, 18 of the 20 GDT isolates most similar to US-22 are heterozygous (341/355) at the microsatellite locus PinfSSR11 where US-22 is homozygous (341/341) (S3 Table). (The other two GDT isolates are 355/355 at this locus.) Thus, the more parsimonious explanation is that US-22 is a parent rather than an unusual sibling, and that the other parent is homozygous (355/355) at this locus.

It is interesting to note that for the microsatellite locus Pi70 we observed a high frequency of the allele 189 within the NYS-2010/11 population. To date we have only found this allele in US-1 and a few Mexican isolates suggesting that a new introduction was also a contributor to this population.

A recombinant population with characteristics similar to the ones observed for the NYS-2010/11 population has been reported in the past in the United States. Gavino et al. [48] reported a group of isolates that were collected in the Columbia basin of Oregon and Washington in 1993 that satisfied the expectations of sexual recombination. This population was ephemeral with the possible exception of US-11 that may have been one of the recombinants and has persisted to the present time as a successful clonal lineage in the United States [19]. There are similarities between the Columbia basin population and the population described here (NYS-2010/11). In both cases opposite mating types were present in proximity; much greater neutral marker diversity was found than has been reported for most other epidemic populations of *P. infestans* in the United States and Canada, and several possible combinations of alleles occurred at many pairs of polymorphic loci.

The likelihood that many different migrations from diverse sources, or that many mutations caused the high degree of genotypic diversity found in the NYS-2010/11 population, seem very low. Migrations have been documented in the past in the United States and Canada but these have typically involved movement of single clonal lineages from known sources [9], [52], [53]. If the NYS-2010/11 isolates represent a migrant population it is reasonable to assume that these isolates arose at a single place and were then dispersed throughout central New York and surrounding areas, possibly via potato seed tubers or tomato transplants. It is unlikely that many diverse mutations occurred in such a short period of time in a single clonal lineage [48]. In a previous study we investigated the diversity within lineages US-22 and US-23 [13] and found that mutations do happen but only in a few loci. It is thus unlikely that many isolates with many diverse mutations would have arisen exclusively in a restricted area of the United States. The mutations known to occur within the US-22 and US-23 lineages did not affect the conclusions derived from the parentage exclusion analysis. Because of the geographical location of the NYS-2010/11 isolates it is likely that they came from the same place. There are five or six states in the northern USA that produce seeds/tubers and all of these states have had late blight. The exact location of the actual plasmogamy events is unknown. Because of the geographically limited occurrence of these diverse GDT individuals, we hypothesize that recombination also occurred in a single location.

The diverse combination of markers used was essential to infer that the NYS-2010/11 population is probably recombinant and that US-22 is a likely parent. The banding patterns for the RFLP assay using the moderately repetitive, polymorphic probe (RG57) as well as the banding patterns for the allozyme *glucose-6-phosphate isomerase* allowed us to identify the diversity and uniqueness among the *P. infestans* isolates tested. The microsatellite profiles permitted us to further discriminate among the isolates, allowed us to eliminate certain genotypes as potential parents for the NYS-2010/11 isolates and further allowed the prediction of potential parental genotypes. The mitochondrial and nuclear genes studied revealed the relatedness among the NYS-2010/11 isolates. The nuclear gene sequences further allowed us to eliminate certain NYS-2010/11 genotypes as possible progeny of US-22 or US-11.

The parentage exclusion analysis left only US-22 as a potential parent from the current dominant lineages. If we assume some mutations, then the US-8 lineage might survive the parentage exclusion analysis. For example it could be possible that a US-8 derivative could have been a parent for the NYS-2010/11 isolates if a mutation occurred that changed either allele (108 or 112) at the D13 locus to 110. However, this assumption seems unlikely since no derivatives of US-8 with a 110 allele at the D13 locus have been found in the United States. Alternatively, if US-8 had a third “null allele” at the D13 locus, it could have donated this allele to the GDT isolates. US-8 is known to have some loci with three alleles – i.e. at locusPinfSSR4 and at *GPI*. Consequently, it is not possible to absolutely eliminate the possibility that US-8 may have a third “null allele” at the D13 locus. Again, this scenario seems highly unlikely given that neither of the other two alleles is present in the GDT isolates.

Our best inference is that the NYS-2010/11 isolates represent a progeny that originated from at least two recombination events. The geographic location(s) of those recombination events remains unknown. The eventual impact of this recombination event cannot be predicted at this moment. The fact that individuals from this event were detected only in 2010 and 2011 and not in 2012 or 2013 suggests that these isolates were not as aggressive or as fit as subsequent dominant clonal lineages. However, the fact that there is now evidence for a second recombinant population of *P. infestans* detected in the USA indicates that sexual recombination is certainly possible, and there is no reason to believe that such populations will not occur in the future. Diligence in monitoring populations might enable the location of a recombination to be identified so that proper mitigation techniques could be applied.

## Supporting Information

S1 Fig **Flow diagram showing how the parentage exclusion analyses were conducted.** A visual parentage exclusion analyses was possible given that there were only 37 unique *Phytophthora infestans* genotypes.
(PDF)

S2 Fig **STRUCTURE analysis of twelve microsatellite loci for** ***Phytophthora infestans*** **in the United States.** Results for K = 2 to K = 5 are shown. Each color represents one population defined by STRUCTURE. Each isolate is represented by a vertical bar, and the length of each colored segment in each vertical bar represents the proportion contributed by the ancestral population. The number of inferred populations based on the *ΔK* method according to Evanno et al. [32] was two. Groups one, two, and three on the x-axis represent an arbitrary classification of isolates into groups according to their occurrence over time in the United States. Group one contained lineages of *P. infestans* that have not been prevalent in the United States for the past 10 years (US-1, US-6, US-7, US-12, US-14, US-16, US-17, and US-19). Group two contained lineages of *P. infestans* that have been dominant in the past five years (US-8, US-11, US-22, US-23 and US-24) or that have been first described during the past five years in the United States (US-20 and US-21). Group three contained the NYS-2010/11 *P. infestans* isolates that are the focus of this study (GDT-01 to GDT-20).
(PDF)

S3 Fig **Bayesian Information Criterion (BIC) values for increasing values of** ***K*** **.** The BIC decreases until *K* = 8 clusters, after which BIC increases. *K* = 8 also matches the smallest BIC, thus 8 clusters were retained.
(PDF)

S4 Fig **MrBayes trees of haplotypes for each locus sequenced.** Loci are **A**. *PITG_11126*, **B**. *PUA*, **C**. *β*-*tubulin*, and **D**. *TRP1* in *Phytophthora infestans*. Haplotypes shown for each branch tip, correspond to those in S5, S6, S7, S8 Tables. Bayesian posterior probabilities are shown above branches and bootstrap support values obtained by maximum likelihood are shown below branches. Values are not shown for branches that had less than 80% probability/support by both methods. Pie charts represent the number of isolates that contain a particular haplotype within each of the three clusters. Clusters were defined based on the occurrence of *P. infestans* isolates over time in the United States. Numbers within parentheses indicate the number of individuals that contain that haplotype.
(PDF)

S1 Table ***Phytophthora infestans*** **isolates used in this study.**
(PDF)

S2 Table **Mating type, and banding patterns for the allozyme** ***glucose-6-phosphate isomerase*** **and for a restriction fragment length polymorphism (RFLP) assay using the RG57 probe for the 20 unique NYS-2010/11 multilocus genotypes.** Polymorphic sites for the RFLP assay using the RG57 probe are highlighted in grey. Seven of the 24 possible combinations are observed within the 20 NYS-2010/11 genotypes.
(PDF)

S3 Table **Microsatellite calls for multilocus genotypes (MLGs) of** ***Phytophthora infestans*** **used in this study.** The first sixteen MLGs in the list are the reference isolates.
(PDF)

S4 Table **Polymorphic sites for a gene coding for a conserved hypothetical protein (** ***PITG_11126*** **) in 35 isolates of** ***Phytophthora infestans*** **.** Isolates highlighted in yellow are those for which US-22 could not be a parent. Inferred haplotypes are identified with the letter H followed by a number. Total length of the sequence is indicated within parentheses.
(PDF)

S5 Table **Polymorphic sites for a gene coding for a conserved hypothetical protein (** ***PUA*** **) in 35 isolates of** ***Phytophthora infestans*** **.** Inferred haplotypes are identified with the letter H followed by a number. Total length of the sequence is indicated within parentheses.
(PDF)

S6 Table **Polymorphic sites for the gene** ***ß-tubulin*** **in 35 isolates of** ***Phytophthora infestans*** **.** Inferred haplotypes are identified with the letter H followed by a number. Total length of the sequence is indicated within parentheses.
(PDF)

S7 Table **Polymorphic sites for the gene coding for the indole-3-glycerolphosphate synthase-** ***N*** **-(5′-phosphoribosyl)anthranilate isomerase (** ***TRP1*** **) in 35 isolates of** ***Phytophthora infestans*** **.** Inferred haplotypes are identified with the letter H followed by a number. Total length of the sequence is indicated within parentheses.
(PDF)

S8 Table **Microsatellite allele names and sizes for the twelve-microsatellite loci used in this study.** Allele sizes can differ slightly from one laboratory to another because of different equipment. In order to compare across locations, the community uses common standards to identify alleles. This sometimes results in an allele name being slightly different from the detected size. This keeps the allele names consistent with earlier publications [26].
(PDF)

S9 Table **Microsatellite calls for multilocus genotypes (MLGs) of** ***Phytophthora infestans*** **used in this study.** Isolates highlighted in grey are those for which US-22 could not be a parent. The alleles shown in red are those not present in US-22.
(PDF)

S10 Table **Microsatellite calls for multilocus genotypes (MLGs) of** ***Phytophthora infestans*** **used in this study.** Isolates highlighted in grey are those for which US-11 could not be a parent. The alleles shown in red are those not present in US-11.
(PDF)

S11 Table **Possible genotypes for the allozyme** ***glucose-6-phosphate isomerase*** **and microsatellite loci of putative parents for the GDT isolates when assuming that lineage US-22 is one of the parental genotypes for these isolates.** Clonal lineage US-22 could not be excluded as a potential parent for 17 of the 20 NYS-2010/11 isolates based on the banding patterns for the allozyme *glucose-6-phosphate isomerase* and microsatellite data. In red we show the alleles that the alternate parent or parents must possess to give rise to the genotypic profiles observed in the NYS-2010/11 isolates when assuming that lineage US-22 is one of the parental genotypes for these isolates.
(PDF)

S12 Table **Possible genotypes of loci** ***PITG_11126*** **,** ***PUA*** **, β-** ***tubulin*** **and** ***TRP1*** **of putative parents for the GDT isolates when assuming that lineage US-22 is one of the parental genotypes for these isolates.** In red we show the alleles that the alternate parent or parents must possess to give rise to the genotypic profiles observed in the NYS-2010/11 isolates when assuming that lineage US-22 is one of the parental genotypes for these isolates.
(PDF)
