# Supplementary material for: An Ephemeral Sexual Population of Phytophthora infestans in the Northeastern United States and Canada
Source: PLoS One. 2014 Dec 31;9(12):e116354. doi: 10.1371/journal.pone.0116354 (PMC4281225; doi:10.1371/journal.pone.0116354)
Supplement: S1 Fig — Flow diagram showing how the parentage exclusion analyses were conducted. A visual parentage exclusion analyses was possible given that there were only 37 unique Phytophthora infestans genotypes. (PDF) [file pone.0116354.s001.pdf]

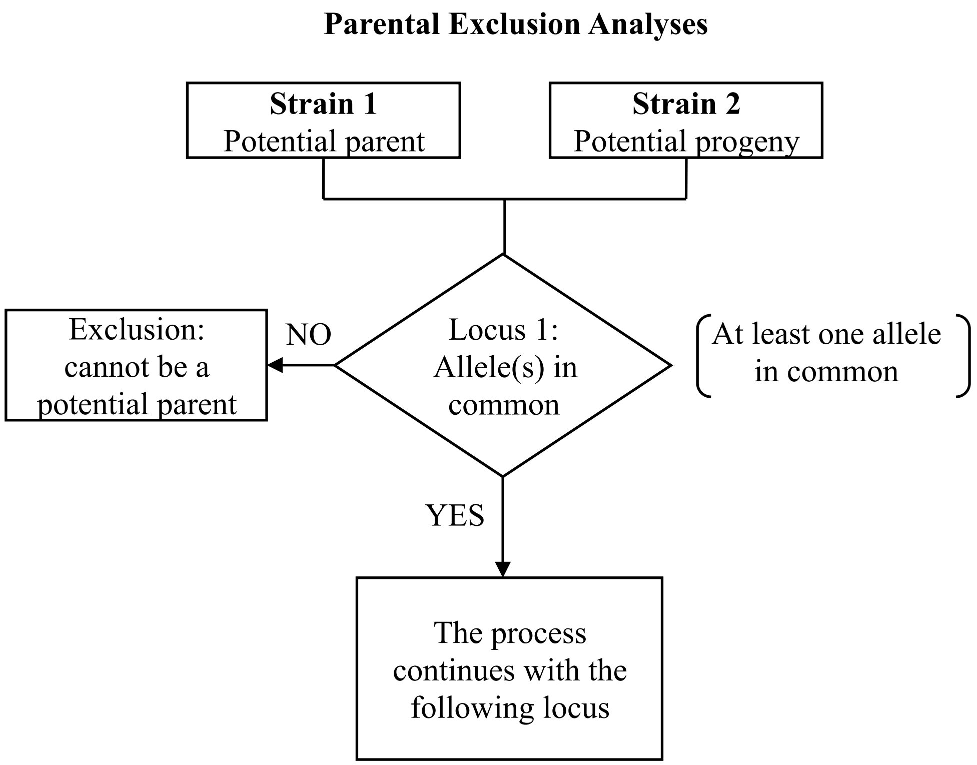

Figure S1. Flow diagram showing how the parentage exclusion analyses were conducted. A visual parentage exclusion analyses was possible given that there were only 37 unique *Phytophthora infestans* genotypes.
