# Supplementary material for: An Ephemeral Sexual Population of Phytophthora infestans in the Northeastern United States and Canada
Source: PLoS One. 2014 Dec 31;9(12):e116354. doi: 10.1371/journal.pone.0116354 (PMC4281225; doi:10.1371/journal.pone.0116354)
Supplement: S2 Fig — STRUCTURE analysis of twelve microsatellite loci for Phytophthora infestans in the United States. Results for K = 2 to K = 5 are shown. Each color represents one population defined by STRUCTURE. Each isolate is represented by a vertical bar, and the length of each colored segment in each vertical bar represents the proportion contributed by the ancestral population. The number of inferred populations based on the ΔK method according to Evanno et al. [32] was two. Groups one, two, and three on the x-axis represent an arbitrary classification of isolates into groups according to their occurrence over time in the United States. Group one contained lineages of P. infestans that have not been prevalent in the United States for the past 10 years (US-1, US-6, US-7, US-12, US-14, US-16, US-17, and US-19). Group two contained lineages of P. infestans that have been dominant in the past five years (US-8, US-11, US-22, US-23 and US-24) or that have been first described during the past five years in the United States (US-20 and US-21). Group three contained the NYS-2010/11 P. infestans isolates that are the focus of this study (GDT-01 to GDT-20). (PDF) [file pone.0116354.s002.pdf]

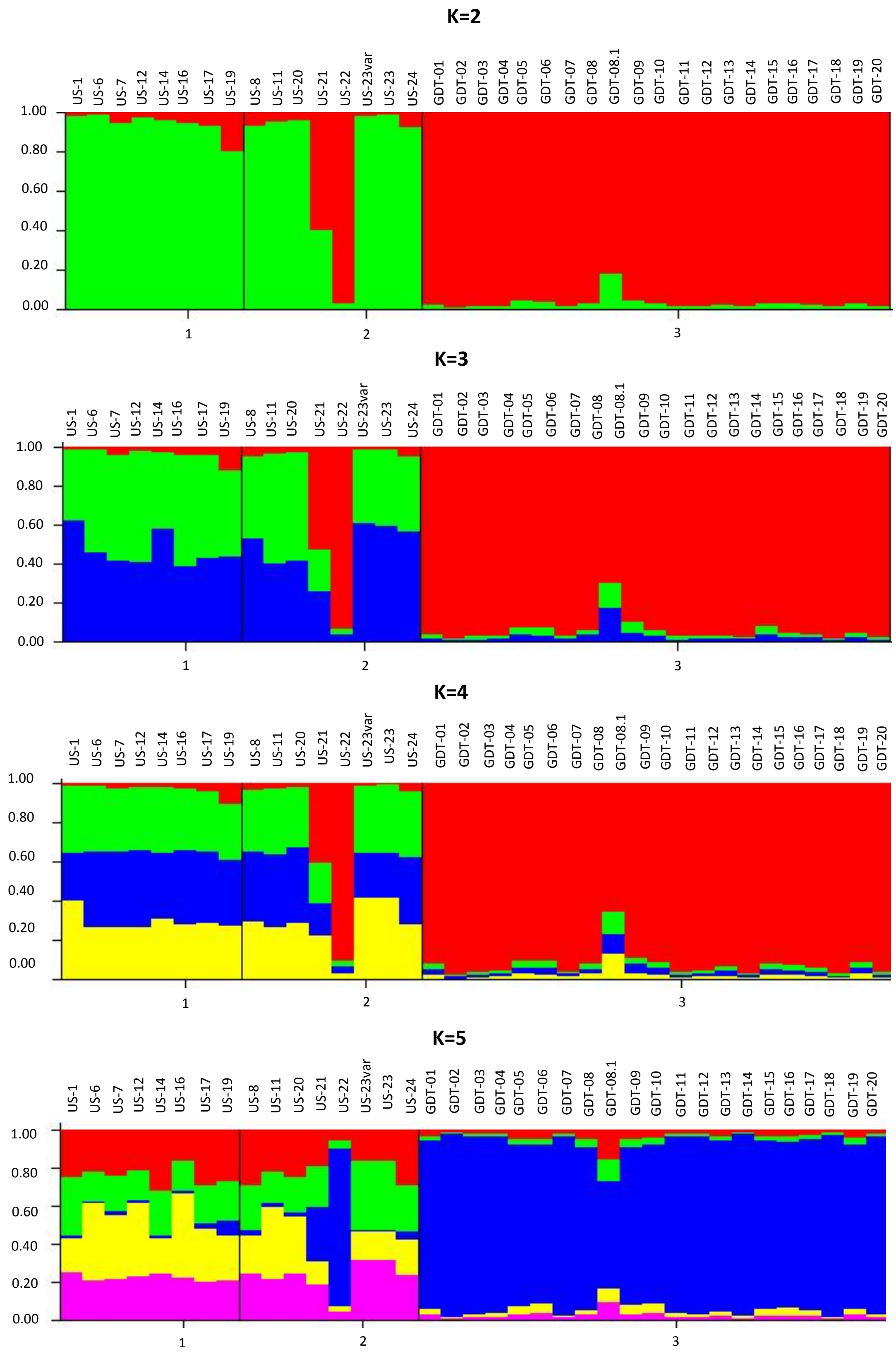
K=2
US-1 US-6 US-7 US-12 US-14 US-16 US-17 US-19 US-8 US-11 US-20 US-21 US-22 US-23var US-23 US-24 GDT-01 GDT-02 GDT-03 GDT-04 GDT-05 GDT-06 GDT-07 GDT-08 GDT-08.1 GDT-09 GDT-10 GDT-11 GDT-12 GDT-13 GDT-14 GDT-15 GDT-16 GDT-17 GDT-18 GDT-19 GDT-20
1.00 0.80 0.60 0.40 0.20 0.00
1 2 3
K=3
US-1 US-6 US-7 US-12 US-14 US-16 US-17 US-19 US-8 US-11 US-20 US-21 US-22 US-23var US-23 US-24 GDT-01 GDT-02 GDT-03 GDT-04 GDT-05 GDT-06 GDT-07 GDT-08 GDT-08.1 GDT-09 GDT-10 GDT-11 GDT-12 GDT-13 GDT-14 GDT-15 GDT-16 GDT-17 GDT-18 GDT-19 GDT-20
1.00 0.80 0.60 0.40 0.20 0.00
1 2 3
K=4
US-1 US-6 US-7 US-12 US-14 US-16 US-17 US-19 US-8 US-11 US-20 US-21 US-22 US-23var US-23 US-24 GDT-01 GDT-02 GDT-03 GDT-04 GDT-05 GDT-06 GDT-07 GDT-08 GDT-08.1 GDT-09 GDT-10 GDT-11 GDT-12 GDT-13 GDT-14 GDT-15 GDT-16 GDT-17 GDT-18 GDT-19 GDT-20
1.00 0.80 0.60 0.40 0.20 0.00
1 2 3
K=5
US-1 US-6 US-7 US-12 US-14 US-16 US-17 US-19 US-8 US-11 US-20 US-21 US-22 US-23var US-23 US-24 GDT-01 GDT-02 GDT-03 GDT-04 GDT-05 GDT-06 GDT-07 GDT-08 GDT-08.1 GDT-09 GDT-10 GDT-11 GDT-12 GDT-13 GDT-14 GDT-15 GDT-16 GDT-17 GDT-18 GDT-19 GDT-20
1.00 0.80 0.60 0.40 0.20 0.00
1 2 3

Figure S2. STRUCTURE analysis of twelve microsatellite loci for *Phytophthora infestans* in the United States. Results for K=2 to K=5 are shown. Each color represents one population defined by STRUCTURE. Each isolate is represented by a vertical bar, and the length of each colored segment in each vertical bar represents the proportion contributed by the ancestral population. The number of inferred populations based on the ΔK method according to Evanno et al. [32] was two. Groups one, two, and three on the x-axis represent an arbitrary classification of isolates into groups according to their occurrence over time in the United States. Group one contained lineages of *P. infestans* that have not been prevalent in the United States for the past 10 years (US-1, US-6, US-7, US-12, US-14, US-16, US-17, and US-19). Group two contained lineages of *P. infestans* that have been dominant in the past five years (US-8, US-11, US-22, US-23 and US-24) or that have been first described during the past five years in the United States (US-20 and US-21). Group three contained the NYS-2010/11 *P. infestans* isolates that are the focus of this study (GDT-01 to GDT-20).
