# Supplementary material for: An Ephemeral Sexual Population of Phytophthora infestans in the Northeastern United States and Canada
Source: PLoS One. 2014 Dec 31;9(12):e116354. doi: 10.1371/journal.pone.0116354 (PMC4281225; doi:10.1371/journal.pone.0116354)
Supplement: S3 Fig — Bayesian Information Criterion (BIC) values for increasing values of K . The BIC decreases until K = 8 clusters, after which BIC increases. K = 8 also matches the smallest BIC, thus 8 clusters were retained. (PDF) [file pone.0116354.s003.pdf]

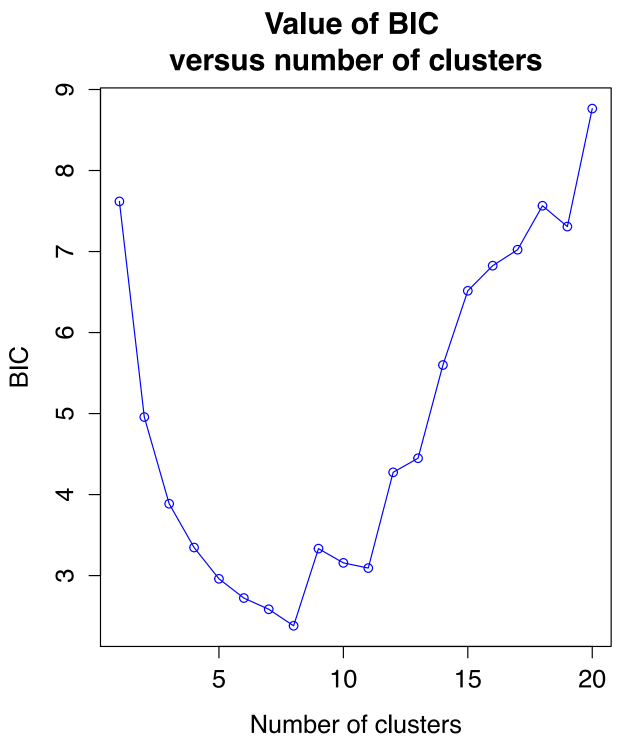

Figure S3. Bayesian Information Criterion (BIC) values for increasing values of *K*.

The BIC decreases until $K = 8$ clusters, after which BIC increases. $K = 8$ also matches the smallest BIC, thus 8 clusters were retained.
