# Supplementary material for: An Ephemeral Sexual Population of Phytophthora infestans in the Northeastern United States and Canada
Source: PLoS One. 2014 Dec 31;9(12):e116354. doi: 10.1371/journal.pone.0116354 (PMC4281225; doi:10.1371/journal.pone.0116354)
Supplement: S4 Fig — MrBayes trees of haplotypes for each locus sequenced. Loci are A. PITG_11126, B. PUA, C. β-tubulin, and D. TRP1 in Phytophthora infestans. Haplotypes shown for each branch tip, correspond to those in S5, S6, S7, S8 Tables. Bayesian posterior probabilities are shown above branches and bootstrap support values obtained by maximum likelihood are shown below branches. Values are not shown for branches that had less than 80% probability/support by both methods. Pie charts represent the number of isolates that contain a particular haplotype within each of the three clusters. Clusters were defined based on the occurrence of P. infestans isolates over time in the United States. Numbers within parentheses indicate the number of individuals that contain that haplotype. (PDF) [file pone.0116354.s004.pdf]

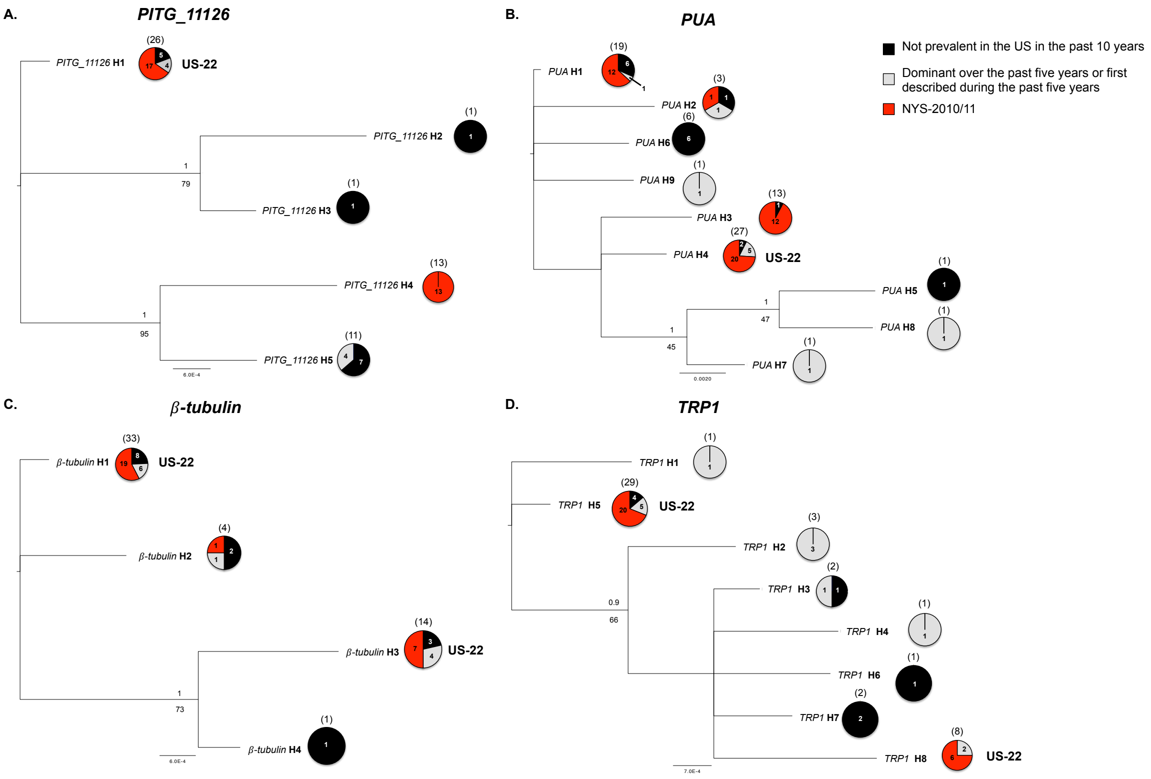

Figure S4. MrBayes trees of haplotypes for each locus sequenced. Loci are **A.** *PITG_11126*, **B.** *PUA*, **C.** *β-tubulin*, and **D.** *TRP1* in *Phytophthora infestans*. Haplotypes shown for each branch tip, correspond to those in Tables S5, S6, S7 and S8. Bayesian posterior probabilities are shown above branches and bootstrap support values obtained by maximum likelihood are shown below branches. Values are not shown for branches that had less than 80% probability/support by both methods. Pie charts represent the number of isolates that contain a particular haplotype within each of the three clusters. Clusters were defined based on the occurrence of *P. infestans* isolates over time in the United States. Numbers within parentheses indicate the number of individuals that contain that haplotype.
