# Supplementary material for: An Ephemeral Sexual Population of Phytophthora infestans in the Northeastern United States and Canada
Source: PLoS One. 2014 Dec 31;9(12):e116354. doi: 10.1371/journal.pone.0116354 (PMC4281225; doi:10.1371/journal.pone.0116354)
Supplement: S1 Table — Phytophthora infestans isolates used in this study. (PDF) [file pone.0116354.s005.pdf]

Table S1. ***Phytophthora infestans*** **isolates used in this study.**

Multilocus Genotype	Isolate	Location County/State abbreviation or Country/Geographic coordinates	Original host	Year	Collector
US-1	SA960008	Mpumalanga, South Africa/ 26ºS 30ºE	Potato	1996	Adele McLeod
US-6	Coffey7629	USA	-	-	Michael D. Coffey
US-7	Coffey7723	USA	-	-	Michael D. Coffey
US-8	US100028	Dufferin/ON Canada/ 44º05'N 80º12'W	Potato	2010	Eugenia Banks
	US110063	Erie/PA/ 42.10ºN 80.10ºW	Potato	2011	Andrew Musa
	US110102	Pasco/WA/ 46º14'19"N 119º6'31"W	Potato	2011	Niklaus Grünwald
US-11	US050007	Benton/OR/ 44º29'25"N 123º25'57"W	Tomato	2005	Melody Putnam
	US110028	Oneida/NY/ 43.24ºN 75.44ºW	Tomato	2011	Jerry Waskiewicz
US-12	US940494	Tompkins/NY/ 42.45ºN 76.47ºW	Tomato	1994	William Fry
US-14	Pi-001-01	MI/ 44ºN 85ºW	-	-	Ken Deahl
US-16	Coffey10112	OR/ 44ºN 120.5ºW	-	1994	Michael D. Coffey
US-17	US970001	Lee/FL/ 26.58ºN 81.92ºW	Tomato	1997	William Fry
US-19	NC09719	NC/ 35.5ºN 80ºW	Tomato	1997	Jean Ristaino
US-20	NC046	NC/ 35.5ºN 80ºW	Tomato	2004	Jean Ristaino
US-21	NC0719	NC/ 35.5ºN 80ºW	Tomato	2007	Jean Ristaino
US-22	US070001	Suffolk/NY/ 40.94ºN 72.68ºW	Tomato	2007	Margaret McGrath
	US090029	Orange/NY/ 41.40ºN 74.31ºW	Tomato	2009	CPDDC

	US090042	Chautuaqua/NY/ 42.30ºN 79.41ºW	Tomato	2009	CPDDC
US-23var	BL2009P4	Blair/PA/ 40.47ºN 78.35ºW	Potato	2009	Ken Deahl
US-23	Pi431	-	-	2013	Dawn Tidd
	Pi432	-	-	2013	Dawn Tidd
	US110017	Suffolk/NY/ 40.94ºN 72.68ºW	Tomato	2011	Margaret McGrath
	US110040	Waukesha/WI/ 43.02ºN 88.31ºW	Tomato	2011	Amanda Gevens
	US110062	Aroostook/ME/ 46.65ºN 68.59ºW	Potato	2011	Steve Johnson
US-24	ND822Pi	ND/ 47ºN 100ºW	Tomato	2009	Gary Secor
	US110004	ND/ 47ºN 100ºW	Potato	2011	Gary Secor
	US110159	Cass/ND/ 46.93ºN 97.25ºW	Potato	2011	Gary Secor
GDT-01	US110057	Ontario/NY/ 42.85ºN 77.29ºW	Tomato	2011	Christine Smart
	US110058	Ontario/NY/ 42.85ºN 77.29ºW	Tomato	2011	Christine Smart
	US110066	Ontario/NY/ 42.85ºN 77.29ºW	Tomato	2011	Christine Smart
	US110073	Wayne/NY/ 43.28ºN 77.05ºW	Potato	2011	Christine Smart
	US110084	Ontario/NY/ 42.85ºN 77.29ºW	Tomato	2011	Christine Smart
	US110135	Ontario/NY/ 42.85ºN 77.29ºW	Nightshade	2011	Christine Smart
GDT-02	US110064	Genesee/NY/ 43.00ºN 78.19ºW	Potato	2011	Christine Smart
GDT-03	US110086	Monroe/NY/ 43.30ºN 77.69ºW	Potato	2011	Christine Smart
GDT-04	US110060	Wyoming/NY/ 42º50’N 78º5’W	Tomato	2011	Don Casiewicz
	US110065	Livingston/NY/ 42.73ºN 77.77ºW	Tomato	2011	Christine Smart
	US110074	Genesee/NY/ 43.00ºN 78.19ºW	Tomato	2011	Christine Smart
	US110075	Genesee/NY/ 43.00ºN 78.19ºW	Tomato	2011	Christine Smart

	US110078	Tompkins/NY/<br>42.45ºN 76.47ºW	Tomato	2011	Keith Perry
	US110094	Tompkins/NY/<br>42.45ºN 76.47ºW	Tomato	2011	Cliff Kraft
GDT-05	US110061	Cayuga/NY/<br>42.94ºN 76.56ºW	Tomato	2011	Sharon Bachman
GDT-06	US110093	Tompkins/NY/<br>42.45ºN 76.47ºW	Tomato	2011	Lisa Hahn
GDT-07	US110072	Genesee/NY/<br>43.00ºN 78.19ºW	Potato	2011	Christine Smart
GDT-08	US110071	Tompkins/NY/<br>42.45ºN 76.47ºW	Tomato	2011	Monica Roth
	US110114	Tompkins/NY/<br>42.45ºN 76.47ºW	Potato	2011	William Fry
	US110128	Tompkins/NY/<br>42.45ºN 76.47ºW	Potato	2011	William Fry
	US110141	Yates/NY/<br>42.64ºN 77.10ºW	Tomato	2011	Abby Seaman
	US110142	Tompkins/NY/<br>42.45ºN 76.47ºW	Tomato	2011	William Fry
GDT-08.1	US110069	Tompkins/NY/<br>42.45ºN 76.47ºW	Tomato	2011	Tom Zitter
GDT-09	US110082	Genesee/NY/<br>43.00ºN 78.19ºW	Tomato	2011	Christine Smart
	US110138	Genesee/NY/<br>43.00ºN 78.19ºW	Tomato	2011	Christine Smart
	US110139	Genesee/NY/<br>43.00ºN 78.19ºW	Tomato	2011	Christine Smart
GDT-10	US110083	Erie/NY/<br>42.75N 78.78ºW	Tomato	2011	Sharon Bachman
GDT-11	US110085	Genesee/NY/<br>43.00ºN 78.19ºW	Nightshade	2011	Christine Smart
GDT-12	US100029	Simcoe/ON Canada/<br>44º1499ºN 79.8632ºW	Potato	2010	Eugenia Banks
GDT-13	US100023	Simcoe/ON Canada/<br>43º35’N 79º44’W	Tomato	2010	Catarina Saude
GDT-14	US110054	Mercer/PA/<br>41.31ºN 80.25ºW	Tomato	2011	Beth Gugino
	US110079	Venango/PA/	Tomato	2011	Beth Gugino

	US110106	41.40ºN 79.76ºW Indiana/PA/ 40.65ºN 79.09ºW	Tomato	2011	Beth Gugino
GDT-15	US100032	Oxford/ON Canada/ 43º14'N 80º36'W	Potato	2010	Eugenia Banks
GDT-16	US100033	Oxford/ON Canada/ 43º14'N 80º36'W	Tomato	2010	Eugenia Banks
GDT-17	US110092	Erie/OH/ 41.51ºN 82.61ºW	Tomato	2011	Sally Miller
GDT-18	US100022	Genesee/NY/ 43.00ºN 78.19ºW	Tomato	2010	Christine Smart
GDT-19	US100019	Erie/NY/ 42.75N 78.78ºW	Tomato	2010	Carol MacNeil
GDT-20	US100034	Niagara/NY/ 43.32ºN 78.79ºW	Tomato	2010	Christy Hoepting

CPDDC: Cornell Plant Disease Diagnostic Clinic
