# Supplementary material for: An Ephemeral Sexual Population of Phytophthora infestans in the Northeastern United States and Canada
Source: PLoS One. 2014 Dec 31;9(12):e116354. doi: 10.1371/journal.pone.0116354 (PMC4281225; doi:10.1371/journal.pone.0116354)
Supplement: S2 Table — Mating type, and banding patterns for the allozyme glucose-6-phosphate isomerase and for a restriction fragment length polymorphism (RFLP) assay using the RG57 probe for the 20 unique NYS-2010/11 multilocus genotypes. Polymorphic sites for the RFLP assay using the RG57 probe are highlighted in grey. Seven of the 24 possible combinations are observed within the 20 NYS-2010/11 genotypes. (PDF) [file pone.0116354.s006.pdf]

Table S2. Mating type, and banding patterns for the allozyme *glucose-6-phosphate isomerase* and for a restriction fragment length polymorphism (RFLP) assay using the RG57 probe for the 20 unique NYS-2010/11 multilocus genotypes. Polymorphic sites for the RFLP assay using the RG57 probe are highlighted in grey. Seven of the 24 possible combinations are observed within the 20 NYS-2010/11 genotypes.

				RG57[e]																					
**MLG[a]**	**MT[b]**	**GPI[c]**	**MH[d]**	**1**	**2**	**3**	**4**	**5**	**6**	**7**	**8**	**9**	**10**	**11**	**12**	**13**	**14**	**15**	**16**	**17**	**18**	**19**	**20**	**21**	**22**	**23**	**24**	**24a**	**25**
US-1	A1	86/100	H22	1	0	1	1	1	0	1	0	1	1	0	0	1	1	0	1	0	0	0	1	1	0	0	1	0	1
US-6	A1	100/100	-	1	0	1	1	1	1	1	0	0	1	0	0	1	1	0	0	0	1	0	1	1	0	0	1	0	1
US-7	A2	100/111	-	1	0	0	1	1	0	0	0	0	1	0	0	1	1	0	1	0	1	0	1	1	0	0	1	0	1
US-8	A2	100/111/122	H20	1	0	0	1	1	0	0	0	0	1	0	0	1	1	0	1	0	0	0	1	1	0	1	1	0	1
US-11	A1	100/100/111	H1	1	0	1	0	1	1	1	0	0	1	0	0	1	1	0	1	0	1	0	1	1	0	0	1	0	1
US-12	A1	100/111	-	1	0	0	0	1	0	0	0	0	1	0	0	1	1	0	0	0	1	0	1	1	0	0	1	0	1
US-14	A2	100/122	-	1	0	0	0	1	0	0	0	0	1	0	0	1	1	0	1	0	0	0	1	1	0	1	1	0	1
US-16	A1	100/111	-	1	0	0	0	1	1	0	0	0	1	0	0	1	1	0	1	0	1	0	1	1	0	0	1	0	1
US-17	A1	100/122	H28	1	0	1	0	0	0	1	0	0	0	0	0	1	1	0	1	0	1	0	1	1	0	0	1	0	1
US-19	A2	100/100	-	1	0	1	0	1	0	1	0	0	0	0	0	1	1	0	1	0	0	0	1	1	0	0	1	0	1
US-20	A2	100/100	-	1	0	1	0	1	0	1	0	0	1	0	0	1	1	0	1	0	1	0	1	1	0	0	1	0	1
US-21	A2	100/122	H20	1	0	0	0	1	0	0	0	0	1	0	0	1	1	0	0	0	0	0	1	1	0	1	1	0	1
US-22	A2	100/122	H20	1	0	0	0	1	0	0	0	0	0	0	0	1	1	0	1	0	0	0	1	1	0	0	1	0	1
US-23[f]	A1	100/100	H25	1	1	0	0	1	1	0	0	0	1	0	0	1	1	0	0	1	0	1*	1	1	1*	0	1	1	1
US-24	A1	100/100	H13	1	0	1	0	1	0	1	0	0	1	0	0	1	1	0	1	0	0	0	1	1	0	1	1	0	1
GDT-01	A1	100/100	H20	1	0	0	0	1	0	0	0	0	0	0	0	1	1	0	**1**	0	**0**	**1**	1	1	**1**	0	1	0	1
GDT-02	A2	111/122	H20	1	0	0	0	1	0	0	0	0	0	0	0	1	1	0	1	0	0	1	1	1	1	0	1	0	1
GDT-03	A2	100/111/122	H20	1	0	0	0	1	0	0	0	0	0	0	0	1	1	0	1	0	0	1	1	1	1	0	1	0	1
GDT-04	A2	100/111	H20	1	0	0	0	1	0	0	0	0	0	0	0	1	1	0	**1**	0	**0**	**0**	1	1	**0**	0	1	0	1
GDT-05	A1	100/111	H20	1	0	0	0	1	0	0	0	0	0	0	0	1	1	0	1	0	0	0	1	1	0	0	1	0	1

GDT-06	A2	100/111	H20	1	0	0	0	1	0	0	0	0	0	0	0	1	1	0	1	0	0	0	1	1	0	0	1	0	1
GDT-07	A2	100/111	H20	1	0	0	0	1	0	0	0	0	0	0	0	1	1	0	**0**	0	**1**	**1**	1	1	**1**	0	1	0	1
GDT-08	A1	100/111	H20	1	0	0	0	1	0	0	0	0	0	0	0	1	1	0	**1**	0	**1**	**1**	1	1	**0**	0	1	0	1
GDT-09	A2	100/122	H20	1	0	0	0	1	0	0	0	0	0	0	0	1	1	0	**1**	0	**1**	**0**	1	1	**0**	0	1	0	1
GDT-10	A1	100/122	H20	1	0	0	0	1	0	0	0	0	0	0	0	1	1	0	**1**	0	**1**	**1**	1	1	**1**	0	1	0	1
GDT-11	A1	111/111	H20	1	0	0	0	0	0	0	0	0	0	0	0	1	1	0	1	0	1	0	1	1	0	0	1	0	1
GDT-12	A1	100/111	H20	1	0	0	0	1	0	0	0	0	0	0	0	1	1	0	1	0	1	1	1	1	0	0	1	0	1
GDT-13	A2	100/111	H20	1	0	0	0	1	0	0	0	0	0	0	0	1	1	0	1	0	1	1	1	1	0	0	1	0	1
GDT-14	A1	111/122	H20	1	0	0	0	1	0	0	0	0	0	0	0	1	1	0	1	0	0	0	1	1	0	0	1	0	1
GDT-15	A2	111/122	H20	1	0	0	0	1	0	0	0	0	0	0	0	1	1	0	1	0	1	0	1	1	0	0	1	0	1
GDT-16	A2	111/122	H20	1	0	0	0	1	0	0	0	0	0	0	0	1	1	0	1	0	1	0	1	1	0	0	1	0	1
GDT-17	A2	100/122	H20	1	0	0	0	1	0	0	0	0	0	0	0	1	1	0	1	0	0	1	1	1	1	0	1	0	1
GDT-18	A2	100/111	H20	1	0	0	0	1	0	0	0	0	0	0	0	1	1	0	**0**	0	**1**	**0**	1	1	**0**	0	1	0	1
GDT-19	A1	100/100	H20	1	0	0	0	1	0	0	0	0	0	0	0	1	1	0	0	0	1	0	1	1	0	0	1	0	1
GDT-20	A1	100/111	H20	1	0	0	0	1	0	0	0	0	0	0	0	1	1	0	0	0	1	0	1	1	0	0	1	0	1

[a] Multilocus genotype
[b] Mating type
[c] Glucose-6-phosphate isomerase
[d] Mitochondrial haplotype
[e] Restriction Fragment Length Polymorphism (RFLP) bands using the RG57 probe
[f] Isolate BL2009 belonging to clonal lineage US-23 lacks band 17
[*] Bands 19 and 22 have not been described in previous reports for US-23 isolate. We decided to include them here because these two bands are seen on all US-23 isolates analyzed.
