# Supplementary material for: An Ephemeral Sexual Population of Phytophthora infestans in the Northeastern United States and Canada
Source: PLoS One. 2014 Dec 31;9(12):e116354. doi: 10.1371/journal.pone.0116354 (PMC4281225; doi:10.1371/journal.pone.0116354)
Supplement: S3 Table — Microsatellite calls for multilocus genotypes (MLGs) of Phytophthora infestans used in this study. The first sixteen MLGs in the list are the reference isolates. (PDF) [file pone.0116354.s007.pdf]

Table S3. Microsatellite calls for multilocus genotypes (MLGs) of *Phytophthora infestans* used in this study. The first 16 MLGs in the list are the reference isolates.

	D13*			PiG11			Pi04			PinfSSR2			Pi70			Pi4B			PinfSSR6			PinfSSR8			Pi02			Pi63			PinfSSR4			PinfSSR11		
MLG	1	2	3	1	2	3	1	2	3	1	2	3	1	2	3	1	2	3	1	2	3	1	2	3	1	2	3	1	2	3	1	2	3	1	2	3
US-1	136	136	0	154	200	0	166	170	0	173	177	0	189	192	0	213	217	0	242	244	0	266	266	0	266	266	0	273	276	279	288	292	0	341	355	0
US-6	144	144	0	156	206	0	166	170	0	173	173	0	192	192	0	213	217	0	244	256	0	264	266	0	258	266	0	279	279	0	290	306	0	341	355	0
US-7	110	110	0	134	156	0	166	170	0	173	173	0	192	192	0	213	213	0	244	244	0	264	266	0	266	268	0	279	279	0	284	298	0	331	341	0
US-8	108	112	0	156	156	0	166	170	0	173	173	0	192	192	0	213	225	0	244	244	0	260	266	0	266	268	0	279	279	0	284	288	294	341	355	0
US-11	110	110	0	130	156	0	166	170	0	173	173	0	192	192	0	213	213	0	244	244	0	264	266	0	258	266	268	279	279	0	284	294	306	331	341	0
US-12	144	148	0	134	156	0	166	170	0	173	173	0	192	192	0	213	213	0	244	256	0	264	264	0	258	266	0	279	279	0	284	294	0	331	355	0
US-14	108	112	0	156	160	0	166	170	0	173	173	0	192	192	0	213	225	0	244	244	0	260	266	0	266	266	0	279	279	0	288	294	0	341	355	0
US-16	110	110	0	156	156	0	166	170	0	173	173	0	192	192	0	213	213	0	256	256	0	264	266	0	258	266	0	279	279	0	284	306	0	341	341	0
US-17	0	0	0	134	156	0	166	170	0	173	173	0	192	192	0	213	213	0	236	244	0	0	0	0	258	266	0	279	279	0	288	288	0	331	355	0
US-19	110	110	0	134	154	0	166	170	0	173	173	0	192	192	0	213	213	0	244	244	0	260	264	0	266	266	0	279	279	0	288	298	0	355	355	0
US-20	108	108	0	134	156	0	166	170	0	173	173	0	192	192	0	213	213	0	244	244	0	264	266	0	258	266	0	279	279	0	284	288	0	355	355	0
US-21	110	110	0	134	134	0	166	170	0	173	173	0	192	192	0	217	217	0	242	244	0	260	260	0	266	268	0	279	279	0	288	286	0	341	341	0
US-22	0	0	0	134	156	0	166	170	0	173	175	0	192	195	0	213	213	0	242	244	0	260	264	0	266	268	0	279	279	0	294	296	0	341	341	0
US-23var	136	136	0	142	156	206	170	170	0	173	175	0	192	192	0	213	217	0	244	244	0	260	266	0	266	268	270	273	279	0	288	294	296	331	341	0
US-23	136	136	0	142	156	206	170	170	0	173	175	0	192	192	0	213	217	0	244	244	0	260	266	0	266	268	270	273	279	0	288	294	0	331	341	0
US-24	108	112	0	156	156	0	166	170	0	173	173	0	192	195	0	217	225	0	244	244	0	260	266	0	266	268	0	279	279	0	284	288	296	341	355	0
GDT-01	110	110	0	134	134	0	166	166	0	173	173	0	192	192	0	213	213	0	242	244	0	260	260	0	258	268	0	279	279	0	288	296	0	341	355	0
GDT-02	0	0	0	134	156	0	166	166	0	173	175	0	189	195	0	213	225	0	242	242	0	260	260	0	258	266	0	279	279	0	284	294	0	341	355	0
GDT-03	110	110	0	134	156	0	166	170	0	173	173	0	189	192	195	213	213	0	242	244	0	260	264	0	258	266	268	279	279	0	288	294	0	341	355	0
GDT-04	110	110	0	134	156	0	166	170	0	173	173	0	189	192	0	213	213	0	242	244	0	260	264	0	258	266	268	279	279	0	284	294	296	341	355	0
GDT-05	0	0	0	156	156	0	166	170	0	173	173	0	192	195	0	213	213	0	242	244	0	260	264	0	258	266	0	279	279	0	288	296	0	341	355	0
GDT-06	110	110	0	156	156	0	166	170	0	173	175	0	192	192	0	213	213	0	242	244	0	260	264	0	258	266	0	279	279	0	284	284	0	341	355	0
GDT-07	0	0	0	156	156	0	166	166	0	173	175	0	192	195	0	213	225	0	242	244	0	260	260	0	258	266	0	279	279	0	288	294	0	341	355	0
GDT-08	0	0	0	134	156	0	166	170	0	173	175	0	189	192	0	213	225	0	242	244	0	260	264	0	266	258	0	279	279	0	288	294	0	341	355	0
GDT-08.1	0	0	0	134	156	0	166	170	0	173	175	0	189	192	0	213	225	0	242	244	0	260	264	0	266	268	0	279	279	0	288	292	294	341	355	0
GDT-09	110	110	0	156	156	0	166	170	0	173	173	0	189	192	0	213	213	0	244	244	0	260	264	0	266	266	0	279	279	0	288	294	0	341	355	0
GDT-10	110	110	0	156	156	0	166	170	0	173	173	0	192	192	0	213	213	0	242	244	0	260	264	0	266	266	0	279	279	0	288	294	0	341	355	0
GDT-11	110	110	0	156	156	0	166	170	0	173	173	0	192	192	0	213	213	0	242	242	0	260	264	0	258	266	0	279	279	0	288	294	0	355	355	0
GDT-12	110	110	0	156	156	0	166	170	0	173	173	0	189	192	0	213	225	0	242	244	0	260	260	0	258	266	0	279	279	0	284	288	0	355	355	0
GDT-13	0	0	0	134	156	0	166	166	0	173	173	0	192	195	0	213	225	0	242	244	0	260	260	0	258	266	0	279	279	0	284	294	0	341	355	0
GDT-14	110	110	0	156	156	0	166	170	0	173	175	0	189	192	0	213	225	0	242	244	0	260	264	0	258	266	268	279	279	0	288	294	296	341	355	0
GDT-15	110	110	0	156	156	0	166	170	0	173	175	0	189	192	0	213	213	0	244	244	0	260	264	0	258	268	0	279	279	0	284	294	0	341	355	0
GDT-16	0	0	0	156	156	0	166	166	0	173	173	0	189	192	0	213	213	0	242	244	0	260	264	0	266	266	0	279	279	0	288	296	0	341	355	0
GDT-17	110	110	0	134	156	0	166	170	0	173	173	0	189	192	0	213	213	0	242	244	0	260	264	0	266	266	0	279	279	0	288	296	0	341	355	0
GDT-18	0	0	0	156	156	0	166	166	0	173	175	0	192	195	0	213	225	0	242	242	0	260	260	0	266	268	0	279	279	0	288	296	0	341	355	0
GDT-19	0	0	0	156	156	0	166	170	0	173	173	0	192	195	0	213	225	0	242	244	0	260	260	0	266	266	0	279	279	0	288	294	0	341	355	0
GDT-20	0	0	0	156	156	0	166	166	0	173	175	0	192	192	0	213	225	0	242	242	0	260	260	0	258	268	0	279	279	0	284	294	0	341	355	0

* A weak 210 allele is observed for US-23 and a weak 230 allele is observed for some GDT isolates.
