# Supplementary material for: An Ephemeral Sexual Population of Phytophthora infestans in the Northeastern United States and Canada
Source: PLoS One. 2014 Dec 31;9(12):e116354. doi: 10.1371/journal.pone.0116354 (PMC4281225; doi:10.1371/journal.pone.0116354)
Supplement: S4 Table — Polymorphic sites for a gene coding for a conserved hypothetical protein ( PITG_11126 ) in 35 isolates of Phytophthora infestans . Isolates highlighted in yellow are those for which US-22 could not be a parent. Inferred haplotypes are identified with the letter H followed by a number. Total length of the sequence is indicated within parentheses. (PDF) [file pone.0116354.s008.pdf]

Table S4. Polymorphic sites for a gene coding for a conserved hypothetical protein (*PITG_11126*) in 35 isolates of *Phytophthora infestans*. Isolates highlighted in yellow are those for which US-22 could not be a parent. Inferred haplotypes are identified with the letter H followed by a number. Total length of the sequence is indicated within parentheses.

	PITG_11126 (776 bp)					
	**49**	**167**	**460**	**549**	**554**	**725**
**US-1**	Y	G	R	T	G	Y
**US-6**	C	G	G	Y	R	C
**US-7**	C	G	G	C	A	C
**US-8**	C	G	G	Y	R	C
**US-11**	C	G	G	C	A	C
**US-12**	C	G	G	Y	R	C
**US-14**	C	G	G	Y	R	C
**US-16**	C	G	G	C	A	C
**US-17**	C	G	G	C	A	C
**US-19**	C	G	G	Y	R	C
**US-20**	C	G	G	C	A	C
**US-21**	C	G	G	T	G	C
**US-22**	**C**	**G**	**G**	**T**	**G**	**C**
**US-23**	-	-	-	-	-	-
**US-24**	C	G	G	Y	R	C
**GDT-01**	C	G	G	T	G	C
**GDT-02**	C	R	G	Y	R	C
**GDT-03**	C	G	G	T	G	C
**GDT-04**	C	A	G	C	A	C
**GDT-05**	C	A	G	C	A	C
**GDT-06**	C	G	G	T	G	C
**GDT-07**	C	G	G	T	G	C
**GDT-08**	C	R	G	Y	R	C
**GDT-09**	C	A	G	C	A	C
**GDT-10**	C	R	G	Y	R	C
**GDT-11**	C	R	G	Y	R	C
**GDT-12**	C	R	G	Y	R	C
**GDT-13**	C	R	G	Y	R	C
**GDT-14**	C	R	G	Y	R	C
**GDT-15**	C	R	G	Y	R	C
**GDT-16**	C	G	G	T	G	C
**GDT-17**	C	G	G	T	G	C
**GDT-18**	C	R	G	Y	R	C
**GDT-19**	C	G	G	T	G	C
**GDT-20**	C	R	G	Y	R	C

R = G/A Y = C/T

**H1**	C	G	G	T	G	C
**H2**	T	G	A	T	G	T
**H3**	T	G	G	T	G	C
**H4**	C	A	G	C	A	C
**H5**	C	G	G	C	A	C
