# Supplementary material for: An Ephemeral Sexual Population of Phytophthora infestans in the Northeastern United States and Canada
Source: PLoS One. 2014 Dec 31;9(12):e116354. doi: 10.1371/journal.pone.0116354 (PMC4281225; doi:10.1371/journal.pone.0116354)
Supplement: S5 Table — Polymorphic sites for a gene coding for a conserved hypothetical protein ( PUA ) in 35 isolates of Phytophthora infestans . Inferred haplotypes are identified with the letter H followed by a number. Total length of the sequence is indicated within parentheses. (PDF) [file pone.0116354.s009.pdf]

Table S5. Polymorphic sites for a gene coding for a conserved hypothetical protein (*PUA*) in 35 isolates of *Phytophthora infestans*. Inferred haplotypes are identified with the letter H followed by a number. Total length of the sequence is indicated within parentheses.

	PUA (609 bp)								
	**35**	**82**	**187**	**381**	**389**	**532**	**555**	**556**	**586**
**US-1**	W	W	Y	G	A	G	Y	W	A
**US-6**	T	T	C	S	A	G	T	T	R
**US-7**	T	T	C	G	M	R	T	T	A
**US-8**	T	T	C	G	C	A	T	T	A
**US-11**	T	T	C	G	A	G	T	T	A
**US-12**	T	T	C	C	A	G	T	T	G
**US-14**	T	T	C	G	C	A	T	T	A
**US-16**	T	T	C	G	A	G	T	T	A
**US-17**	T	T	C	G	A	G	T	T	A
**US-19**	T	T	C	S	A	G	T	T	R
**US-20**	T	T	C	G	C	A	T	T	A
**US-21**	W	W	Y	S	M	R	T	T	A
**US-22**	**T**	**T**	**C**	**G**	**C**	**A**	**T**	**T**	**A**
**US-23**	W	W	Y	S	M	R	Y	T	A
**US-24**	T	T	C	G	C	A	T	T	A
**GDT-01**	T	T	C	G	M	R	T	T	A
**GDT-02**	T	T	C	G	M	R	T	T	A
**GDT-03**	T	T	C	G	M	R	T	T	A
**GDT-04**	T	T	C	G	C	A	T	T	A
**GDT-05**	T	T	C	G	M	R	T	T	A
**GDT-06**	T	T	C	G	C	A	T	T	A
**GDT-07**	T	T	C	G	M	R	T	T	A
**GDT-08**	T	T	C	G	M	R	T	T	A
**GDT-09**	T	T	C	G	M	R	T	T	A
**GDT-10**	T	T	C	G	C	A	T	T	A
**GDT-11**	T	T	C	G	C	A	T	T	A
**GDT-12**	T	T	C	G	M	R	T	T	A
**GDT-13**	T	T	C	G	M	R	T	T	A
**GDT-14**	T	T	C	G	C	A	T	T	A
**GDT-15**	T	T	C	G	C	A	T	T	A
**GDT-16**	T	T	C	G	C	A	T	T	A
**GDT-17**	T	T	C	G	C	A	T	T	A
**GDT-18**	T	T	C	G	M	R	T	T	A
**GDT-19**	T	T	C	G	M	R	T	T	A
**GDT-20**	T	T	C	G	M	R	T	T	A
M = C/A	R = G/A	S = C/G	W = A/T	Y = C/T					
**H1**	T	T	C	G	A	G	T	T	A
**H2**	T	T	C	C	A	G	T	T	G
**H3**	T	T	C	G	A	A	T	T	A
**H4**	T	T	C	G	C	A	T	T	A
**H5**	A	A	T	G	A	G	C	T	A
**H6**	T	T	C	G	A	G	T	A	A
**H7**	A	A	C	G	C	A	T	T	A
**H8**	A	A	T	C	A	A	T	T	A
**H9**	T	T	C	G	C	G	C	T	A
