# Supplementary material for: An Ephemeral Sexual Population of Phytophthora infestans in the Northeastern United States and Canada
Source: PLoS One. 2014 Dec 31;9(12):e116354. doi: 10.1371/journal.pone.0116354 (PMC4281225; doi:10.1371/journal.pone.0116354)
Supplement: S6 Table — Polymorphic sites for the gene ß-tubulin in 35 isolates of Phytophthora infestans . Inferred haplotypes are identified with the letter H followed by a number. Total length of the sequence is indicated within parentheses. (PDF) [file pone.0116354.s010.pdf]

Table S6. Polymorphic sites for the gene *ß-tubulin* in 35 isolates of *Phytophthora infestans*. Inferred haplotypes are identified with the letter H followed by a number. Total length of the sequence is indicated within parentheses.

	ß-tubulin (883 bp)		
	**583**	**808**	**829**
**US-1**	C	C	T
**US-6**	C	Y	T
**US-7**	Y	C	Y
**US-8**	Y	C	Y
**US-11**	Y	Y	Y
**US-12**	C	C	T
**US-14**	Y	C	Y
**US-16**	C	Y	T
**US-17**	C	C	T
**US-19**	Y	C	Y
**US-20**	C	Y	T
**US-21**	C	C	T
**US-22**	**Y**	**C**	**Y**
**US-23**	Y	C	Y
**US-24**	Y	C	Y
**GDT-01**	C	Y	T
**GDT-02**	C	C	T
**GDT-03**	C	C	T
**GDT-04**	T	C	Y
**GDT-05**	C	C	T
**GDT-06**	C	C	T
**GDT-07**	C	C	T
**GDT-08**	Y	C	Y
**GDT-09**	C	C	T
**GDT-10**	C	C	T
**GDT-11**	Y	C	Y
**GDT-12**	C	C	T
**GDT-13**	C	C	T
**GDT-14**	Y	C	Y
**GDT-15**	C	C	T
**GDT-16**	Y	C	Y
**GDT-17**	Y	C	Y
**GDT-18**	Y	C	Y
**GDT-19**	C	C	T
**GDT-20**	C	C	T

Y = C/T

**H1**	C	C	T
**H2**	C	T	T
**H3**	T	C	C
**H4**	T	C	T
