# Supplementary material for: An Ephemeral Sexual Population of Phytophthora infestans in the Northeastern United States and Canada
Source: PLoS One. 2014 Dec 31;9(12):e116354. doi: 10.1371/journal.pone.0116354 (PMC4281225; doi:10.1371/journal.pone.0116354)
Supplement: S7 Table — Polymorphic sites for the gene coding for the indole-3-glycerolphosphate synthase- N -(5′-phosphoribosyl)anthranilate isomerase ( TRP1 ) in 35 isolates of Phytophthora infestans . Inferred haplotypes are identified with the letter H followed by a number. Total length of the sequence is indicated within parentheses. (PDF) [file pone.0116354.s011.pdf]

Table S7. Polymorphic sites for the gene coding for the indole-3-glycerolphosphate synthase-*N*-(5'-phosphoribosyl)anthranilate isomerase (*TRP1*) in 35 isolates of *Phytophthora infestans*. Inferred haplotypes are identified with the letter H followed by a number. Total length of the sequence is indicated within parentheses.

	TRP1 (824 bp)					
	**51**	**78**	**132**	**563**	**614**	**714**
**US-1**	R	A	R	R	T	G
**US-6**	G	A	A	A	W	G
**US-7**	G	A	G	G	T	G
**US-8**	G	A	G	G	T	G
**US-11**	G	R	R	R	T	G
**US-12**	G	A	R	R	W	G
**US-14**	G	A	G	G	T	G
**US-16**	G	A	R	R	T	G
**US-17**	G	A	R	R	T	G
**US-19**	G	A	R	R	T	G
**US-20**	G	A	R	R	T	G
**US-21**	G	A	G	R	T	G
**US-22**	**G**	**A**	**R**	**G**	**T**	**G**
**US-23**	G	A	R	R	T	R
**US-24**	G	A	R	G	T	G
**GDT-01**	G	A	G	G	T	G
**GDT-02**	G	A	R	G	T	G
**GDT-03**	G	A	G	G	T	G
**GDT-04**	G	A	G	G	T	G
**GDT-05**	G	A	R	G	T	G
**GDT-06**	G	A	G	G	T	G
**GDT-07**	G	A	R	G	T	G
**GDT-08**	G	A	G	G	T	G
**GDT-09**	G	A	G	G	T	G
**GDT-10**	G	A	R	G	T	G
**GDT-11**	G	A	G	G	T	G
**GDT-12**	G	A	G	G	T	G
**GDT-13**	G	A	G	G	T	G
**GDT-14**	G	A	G	G	T	G
**GDT-15**	G	A	R	G	T	G
**GDT-16**	G	A	G	G	T	G
**GDT-17**	G	A	G	G	T	G
**GDT-18**	G	A	G	G	T	G
**GDT-19**	G	A	R	G	T	G
**GDT-20**	G	A	G	G	T	G

R = G/A W = A/T

**H1**	G	A	G	G	T	A
**H2**	G	A	G	A	T	G
**H3**	G	A	A	A	T	G
**H4**	G	G	A	A	T	G
**H5**	G	A	G	G	T	G
**H6**	A	A	A	A	T	G
**H7**	G	A	A	A	A	G
**H8**	G	A	A	G	T	G
