# Supplementary material for: An Ephemeral Sexual Population of Phytophthora infestans in the Northeastern United States and Canada
Source: PLoS One. 2014 Dec 31;9(12):e116354. doi: 10.1371/journal.pone.0116354 (PMC4281225; doi:10.1371/journal.pone.0116354)
Supplement: S8 Table — Microsatellite allele names and sizes for the twelve-microsatellite loci used in this study. Allele sizes can differ slightly from one laboratory to another because of different equipment. In order to compare across locations, the community uses common standards to identify alleles. This sometimes results in an allele name being slightly different from the detected size. This keeps the allele names consistent with earlier publications [26]. (PDF) [file pone.0116354.s012.pdf]

Table S8. Microsatellite allele names and sizes for the twelve-microsatellite loci used in this study. Allele sizes can differ slightly from one laboratory to another because of different equipment. In order to compare across locations, the community uses common standards to identify alleles. This sometimes results in an allele name being slightly different from the detected size. This keeps the allele names consistent with earlier publications [26].

D13	**Allele name**	**108**	**110**	**112**	**136**	**144**	**148**		
	Allele size	106	108	110	135	143	148		
PiG11	**Allele name**	**130**	**134**	**142**	**154**	**156**	**160**	**200**	**206**
	Allele size	130	134	142	155	157	161	200	206
Pi04	**Allele name**	**166**	**170**						
	Allele size	171	175						
SSR2	**Allele name**	**173**	**175**	**177**					
	Allele size	173	175	177					
Pi70	**Allele name**	**189**	**192**	**195**					
	Allele size	188	191	194					
Pi4B	**Allele name**	**213**	**217**	**225**					
	Allele size	216	220	228					
SSR6	**Allele name**	**236**	**242**	**244**	**256**				
	Allele size	238	244	246	258				
SSR8	**Allele name**	**260**	**264**	**266**					
	Allele size	262	266	268					
Pi02	**Allele name**	**258**	**266**	**268**	**270**				
	Allele size	260	268	270	272				
Pi63	**Allele name**	**273**	**276**	**279**					
	Allele size	272	275	280					
SSR4	**Allele name**	**284**	**288**	**290**	**292**	**294**	**296**	**298**	**306**
	Allele size	284	288	290	292	294	296	298	306
SSR11	**Allele name**	**331**	**341**	**355**					
	Allele size	331	341	356					
