# Supplementary material for: An Ephemeral Sexual Population of Phytophthora infestans in the Northeastern United States and Canada
Source: PLoS One. 2014 Dec 31;9(12):e116354. doi: 10.1371/journal.pone.0116354 (PMC4281225; doi:10.1371/journal.pone.0116354)
Supplement: S9 Table — Microsatellite calls for multilocus genotypes (MLGs) of Phytophthora infestans used in this study. Isolates highlighted in grey are those for which US-22 could not be a parent. The alleles shown in red are those not present in US-22. (PDF) [file pone.0116354.s013.pdf]

Table S9. Microsatellite calls for multilocus genotypes (MLGs) of *Phytophthora infestans* used in this study. Isolates highlighted in grey are those for which US-22 could not be a parent. The alleles shown in red are those not present in US-22.

	D13			PiG11			Pi04			PinfSSR2			Pi70			Pi4B			PinfSSR6			PinfSSR8			Pi02			Pi63			PinfSSR4			PinfSSR11		
**MLG**	1	2	3	1	2	3	1	2	3	1	2	3	1	2	3	1	2	3	1	2	3	1	2	3	1	2	3	1	2	3	1	2	3	1	2	3
**US-22**	**0**	**0**	**0**	**134**	**156**	**0**	**166**	**170**	**0**	**173**	**175**	**0**	**192**	**195**	**0**	**213**	**213**	**0**	**242**	**244**	**0**	**260**	**264**	**0**	**266**	**268**	**0**	**279**	**279**	**0**	**294**	**296**	**0**	**341**	**341**	**0**
**GDT-01**	110	110	0	134	134	0	166	166	0	173	173	0	192	192	0	213	213	0	242	244	0	260	260	0	258	268	0	279	279	0	288	296	0	341	355	0
**GDT-02**	0	0	0	134	156	0	166	166	0	173	175	0	189	195	0	213	225	0	242	242	0	260	260	0	258	266	0	279	279	0	284	294	0	341	355	0
**GDT-03**	110	110	0	134	156	0	166	170	0	173	173	0	189	192	195	213	213	0	242	244	0	260	264	0	258	266	268	279	279	0	288	294	0	341	355	0
**GDT-04**	110	110	0	134	156	0	166	170	0	173	173	0	189	192	0	213	213	0	242	244	0	260	264	0	258	266	268	279	279	0	284	294	296	341	355	0
**GDT-05**	0	0	0	156	156	0	166	170	0	173	173	0	192	195	0	213	213	0	242	244	0	260	264	0	258	266	0	279	279	0	288	296	0	341	355	0
GDT-06	110	110	0	156	156	0	166	170	0	173	175	0	192	192	0	213	213	0	242	244	0	260	264	0	258	266	0	279	279	0	**284**	**284**	**0**	341	355	0
**GDT-07**	0	0	0	156	156	0	166	166	0	173	175	0	192	195	0	213	225	0	242	244	0	260	260	0	258	266	0	279	279	0	288	294	0	341	355	0
**GDT-08**	0	0	0	134	156	0	166	170	0	173	175	0	189	192	0	213	225	0	242	244	0	260	264	0	266	258	0	279	279	0	288	294	0	341	355	0
**GDT-08.1**	0	0	0	134	156	0	166	170	0	173	175	0	189	192	0	213	225	0	242	244	0	260	264	0	266	268	0	279	279	0	288	292	294	341	355	0
**GDT-09**	110	110	0	156	156	0	166	170	0	173	173	0	189	192	0	213	213	0	244	244	0	260	264	0	266	266	0	279	279	0	288	294	0	341	355	0
**GDT-10**	110	110	0	156	156	0	166	170	0	173	173	0	192	192	0	213	213	0	242	244	0	260	264	0	266	266	0	279	279	0	288	294	0	341	355	0
GDT-11	110	110	0	156	156	0	166	170	0	173	173	0	192	192	0	213	213	0	242	242	0	260	264	0	258	266	0	279	279	0	288	294	0	**355**	**355**	**0**
GDT-12	110	110	0	156	156	0	166	170	0	173	173	0	189	192	0	213	225	0	242	244	0	260	260	0	258	266	0	279	279	0	**284**	**288**	**0**	**355**	**355**	**0**
**GDT-13**	0	0	0	134	156	0	166	166	0	173	173	0	192	195	0	213	225	0	242	244	0	260	260	0	258	266	0	279	279	0	284	294	0	341	355	0
**GDT-14**	110	110	0	156	156	0	166	170	0	173	175	0	189	192	0	213	225	0	242	244	0	260	264	0	258	266	268	279	279	0	288	294	296	341	355	0
**GDT-15**	110	110	0	156	156	0	166	170	0	173	175	0	189	192	0	213	213	0	244	244	0	260	264	0	258	268	0	279	279	0	284	294	0	341	355	0
**GDT-16**	0	0	0	156	156	0	166	166	0	173	173	0	189	192	0	213	213	0	242	244	0	260	264	0	266	266	0	279	279	0	288	296	0	341	355	0
**GDT-17**	110	110	0	134	156	0	166	170	0	173	173	0	189	192	0	213	213	0	242	244	0	260	264	0	266	266	0	279	279	0	288	296	0	341	355	0
**GDT-18**	0	0	0	156	156	0	166	166	0	173	175	0	192	195	0	213	225	0	242	242	0	260	260	0	266	268	0	279	279	0	288	296	0	341	355	0
**GDT-19**	0	0	0	156	156	0	166	170	0	173	173	0	192	195	0	213	225	0	242	244	0	260	260	0	266	266	0	279	279	0	288	294	0	341	355	0
**GDT-20**	0	0	0	156	156	0	166	166	0	173	175	0	192	192	0	213	225	0	242	242	0	260	260	0	258	268	0	279	279	0	284	294	0	341	355	0
