# Supplementary material for: An Ephemeral Sexual Population of Phytophthora infestans in the Northeastern United States and Canada
Source: PLoS One. 2014 Dec 31;9(12):e116354. doi: 10.1371/journal.pone.0116354 (PMC4281225; doi:10.1371/journal.pone.0116354)
Supplement: S10 Table — Microsatellite calls for multilocus genotypes (MLGs) of Phytophthora infestans used in this study. Isolates highlighted in grey are those for which US-11 could not be a parent. The alleles shown in red are those not present in US-11. (PDF) [file pone.0116354.s014.pdf]

Table S10. Microsatellite calls for multilocus genotypes (MLGs) of *Phytophthora infestans* used in this study. Isolates highlighted in grey are those for which US-11 could not be a parent. The alleles shown in red are those not present in US-11.

	D13			PiG11			Pi04			PinfSSR2			Pi70			Pi4B			PinfSSR6			PinfSSR8			Pi02			Pi63			PinfSSR4			PinfSSR11		
**MLG**	1	2	3	1	2	3	1	2	3	1	2	3	1	2	3	1	2	3	1	2	3	1	2	3	1	2	3	1	2	3	1	2	3	1	2	3
**US-11**	**110**	**110**	**0**	**130**	**156**	**0**	**166**	**170**	**0**	**173**	**173**	**0**	**192**	**192**	**0**	**213**	**213**	**0**	**244**	**244**	**0**	**264**	**266**	**0**	**258**	**266**	**268**	**279**	**279**	**0**	**284**	**294**	**306**	**331**	**341**	**0**
GDT-01	110	110	0	**134**	**134**	**0**	166	166	0	173	173	0	192	192	0	213	213	0	242	244	0	**260**	**260**	**0**	258	268	0	279	279	0	**288**	**296**	**0**	341	355	0
GDT-02	**0**	**0**	**0**	134	156	0	166	166	0	173	175	0	**189**	**195**	**0**	213	225	0	**242**	**242**	**0**	**260**	**260**	**0**	258	266	0	279	279	0	284	294	0	341	355	0
**GDT-03**	110	110	0	134	156	0	166	170	0	173	173	0	189	192	195	213	213	0	242	244	0	260	264	0	258	266	268	279	279	0	288	294	0	341	355	0
**GDT-04**	110	110	0	134	156	0	166	170	0	173	173	0	189	192	0	213	213	0	242	244	0	260	264	0	258	266	268	279	279	0	284	294	296	341	355	0
GDT-05	**0**	**0**	**0**	156	156	0	166	170	0	173	173	0	192	195	0	213	213	0	242	244	0	260	264	0	258	266	0	279	279	0	**288**	**296**	**0**	341	355	0
**GDT-06**	110	110	0	156	156	0	166	170	0	173	175	0	192	192	0	213	213	0	242	244	0	260	264	0	258	266	0	279	279	0	284	284	0	341	355	0
GDT-07	**0**	**0**	**0**	156	156	0	166	166	0	173	175	0	192	195	0	213	225	0	242	244	0	**260**	**260**	**0**	258	266	0	279	279	0	288	294	0	341	355	0
GDT-08	**0**	**0**	**0**	134	156	0	166	170	0	173	175	0	189	192	0	213	225	0	242	244	0	260	264	0	266	258	0	279	279	0	288	294	0	341	355	0
GDT-08.1	**0**	**0**	**0**	134	156	0	166	170	0	173	175	0	189	192	0	213	225	0	242	244	0	260	264	0	266	268	0	279	279	0	288	292	294	341	355	0
**GDT-09**	110	110	0	156	156	0	166	170	0	173	173	0	189	192	0	213	213	0	244	244	0	260	264	0	266	266	0	279	279	0	288	294	0	341	355	0
**GDT-10**	110	110	0	156	156	0	166	170	0	173	173	0	192	192	0	213	213	0	242	244	0	260	264	0	266	266	0	279	279	0	288	294	0	341	355	0
GDT-11	110	110	0	156	156	0	166	170	0	173	173	0	192	192	0	213	213	0	**242**	**242**	**0**	260	264	0	258	266	0	279	279	0	288	294	0	**355**	**355**	**0**
GDT-12	110	110	0	156	156	0	166	170	0	173	173	0	189	192	0	213	225	0	242	244	0	**260**	**260**	**0**	258	266	0	279	279	0	284	288	0	**355**	**355**	**0**
GDT-13	**0**	**0**	**0**	134	156	0	166	166	0	173	173	0	192	195	0	213	225	0	242	244	0	**260**	**260**	**0**	258	266	0	279	279	0	284	294	0	341	355	0
**GDT-14**	110	110	0	156	156	0	166	170	0	173	175	0	189	192	0	213	225	0	242	244	0	260	264	0	258	266	268	279	279	0	288	294	296	341	355	0
**GDT-15**	110	110	0	156	156	0	166	170	0	173	175	0	189	192	0	213	213	0	244	244	0	260	264	0	258	268	0	279	279	0	284	294	0	341	355	0
GDT-16	**0**	**0**	**0**	156	156	0	166	166	0	173	173	0	189	192	0	213	213	0	242	244	0	260	264	0	266	266	0	279	279	0	**288**	**296**	**0**	341	355	0
GDT-17	110	110	0	134	156	0	166	170	0	173	173	0	189	192	0	213	213	0	242	244	0	260	264	0	266	266	0	279	279	0	**288**	**296**	**0**	341	355	0
GDT-18	**0**	**0**	**0**	156	156	0	166	166	0	173	175	0	192	195	0	213	225	0	**242**	**242**	**0**	**260**	**260**	**0**	266	268	0	279	279	0	**288**	**296**	**0**	341	355	0
GDT-19	**0**	**0**	**0**	156	156	0	166	170	0	173	173	0	192	195	0	213	225	0	242	244	0	**260**	**260**	**0**	266	266	0	279	279	0	288	294	0	341	355	0
GDT-20	**0**	**0**	**0**	156	156	0	166	166	0	173	175	0	192	192	0	213	225	0	**242**	**242**	**0**	**260**	**260**	**0**	258	268	0	279	279	0	284	294	0	341	355	0
