# Supplementary material for: An Ephemeral Sexual Population of Phytophthora infestans in the Northeastern United States and Canada
Source: PLoS One. 2014 Dec 31;9(12):e116354. doi: 10.1371/journal.pone.0116354 (PMC4281225; doi:10.1371/journal.pone.0116354)
Supplement: S11 Table — Possible genotypes for the allozyme glucose-6-phosphate isomerase and microsatellite loci of putative parents for the GDT isolates when assuming that lineage US-22 is one of the parental genotypes for these isolates. Clonal lineage US-22 could not be excluded as a potential parent for 17 of the 20 NYS-2010/11 isolates based on the banding patterns for the allozyme glucose-6-phosphate isomerase and microsatellite data. In red we show the alleles that the alternate parent or parents must possess to give rise to the genotypic profiles observed in the NYS-2010/11 isolates when assuming that lineage US-22 is one of the parental genotypes for these isolates. (PDF) [file pone.0116354.s015.pdf]

Table S11. Possible genotypes for the allozyme *glucose-6-phosphate isomerase* and microsatellite loci of putative parents for the GDT isolates when assuming that lineage US-22 is one of the parental genotypes for these isolates. Clonal lineage US-22 could not be excluded as a potential parent for 17 of the 20 NYS-2010/11 isolates based on the banding patterns for the allozyme *glucose-6-phosphate isomerase* and microsatellite data. In red we show the alleles that the alternate parent or parents must possess to give rise to the genotypic profiles observed in the NYS-2010/11 isolates when assuming that lineage US-22 is one of the parental genotypes for these isolates.

Alleles present	GPI			Mating type	Microsatellite loci											
					D13	PiG11	Pi04	PinfSSR2	Pi70	Pi4B	PinfSSR6	PinfSSR8	Pi02	Pi63	PinfSSR4	PinfSSR11
**US-22**	100		122	A2	0	134/156	166/170	173/175	192/195	213	242/244	260/264	266/268	279	294/296	341
**GDTs**	100	111	122	A1/A2	0/110	134/156	166/170	173/175	189/192/195	213/225	242/244	260/264	258/266/268	279	284/288/294/296	341/355
**Other parent(s)**		**111**		**A1**	**110**	134/156	166/170	173/175	**189**	**225**	242/244	260/264	**258**	279	**284/288**	**355**
