# Supplementary material for: An Ephemeral Sexual Population of Phytophthora infestans in the Northeastern United States and Canada
Source: PLoS One. 2014 Dec 31;9(12):e116354. doi: 10.1371/journal.pone.0116354 (PMC4281225; doi:10.1371/journal.pone.0116354)
Supplement: S12 Table — Possible genotypes of loci PITG_11126 , PUA , β- tubulin and TRP1 of putative parents for the GDT isolates when assuming that lineage US-22 is one of the parental genotypes for these isolates. In red we show the alleles that the alternate parent or parents must possess to give rise to the genotypic profiles observed in the NYS-2010/11 isolates when assuming that lineage US-22 is one of the parental genotypes for these isolates. (PDF) [file pone.0116354.s016.pdf]

Table S12. Possible genotypes of loci *PITG_11126*, *PUA*, β-*tubulin* and *TRP1* of putative parents for the GDT isolates when assuming that lineage US-22 is one of the parental genotypes for these isolates. In red we show the alleles that the alternate parent or parents must possess to give rise to the genotypic profiles observed in the NYS-2010/11 isolates when assuming that lineage US-22 is one of the parental genotypes for these isolates.

	***PITG_11126* (776 bp)**						***PUA* (609 bp)**										***β-tubulin* (883 bp)**			***TRP1* (824 bp)**					
**Polymorphic site / Alleles present**	49	167	460	549	554	725	35	82	187	381	382	389	532	555	556	586	583	808	829	51	78	132	563	614	714
**US-22**	C	G	G	T	G	C	T	T	C	G	G	C	A	T	T	A	Y	C	Y	G	A	R	G	T	G
**GDTs**	C	G/R/A	G	T/Y/C	G/R/A	C	T	T	C	G	G	C/M	A/R	T	T	T	C/Y/T	C/Y	T/Y	G	A	G/R	G	T	G
**Other parent(s)**	C	G/R/A	G	T/Y/C	G/R/A	C	T	T	C	G	G	C/M/A	G/R/A	T	T	T	C/Y/T	C/Y/T	C/Y/T	G	A	G/R/A	G	T	G
